# The protein deacetylase HDAC10 controls DNA replication in malignant lymphoid cells

**DOI:** 10.1038/s41375-025-02612-8

**Published:** 2025-04-29

**Authors:** Andreas O. Mieland, Giuseppe Petrosino, Mario Dejung, Jia-Xuan Chen, Amitkumar Fulzele, Fereshteh Mahmoudi, Jia-Wey Tu, Al-Hassan M. Mustafa, Yanira Zeyn, Christoph Hieber, Matthias Bros, Tina M. Schnöder, Florian H. Heidel, Sara Najafi, Ina Oehme, Ilse Hofmann, Mike Schutkowski, Sebastian Hilscher, Christian Kosan, Falk Butter, Sanil Bhatia, Wolfgang Sippl, Oliver H. Krämer

**Affiliations:** 1 Institute of Toxicology, Mainz University Medical Center, Mainz, Germany; 2 Institute of Molecular Biology (IMB), Core Facility Bioinformatics, Mainz, Germany; 3 Institute of Molecular Biology (IMB), Core Facility Proteomics, Mainz, Germany; 4 Department of Medicinal Chemistry, Institute of Pharmacy, Martin-Luther-University of Halle-Wittenberg, 06120 Halle (Saale), Germany; 5 Department of Pediatric Oncology, Hematology and Clinical Immunology, Medical Faculty, Heinrich-Heine-University Düsseldorf, 40225 Düsseldorf, Germany; 6 Department of Zoology, Faculty of Science, Aswan University, Aswan, Egypt; 7 Department of Dermatology, University Medical Center Mainz, Mainz, Germany; 8 Hematology, Hemostasis, Oncology and Stem Cell Transplantation, Hannover MedicalSchool (MHH), Hannover, Germany; 9 Leibniz Institute on Aging, Fritz-Lipmann-Institute, Jena, Germany; 10 Hopp Children’s Cancer Center Heidelberg (KiTZ), 69120 Heidelberg, Germany; 11 National Center for Tumor Diseases Heidelberg, 69120 Heidelberg, Germany; 12 Clinical Cooperation Unit Pediatric Oncology, German Cancer Research Center (DKFZ) and German Cancer Consortium (DKTK), 69120 Heidelberg, Germany; 13 Core Facility Antibodies, German Cancer Research Center (DKFZ), Heidelberg, Germany; 14 Institute of Biochemistry and Biotechnology, Martin Luther University Halle-Wittenberg, Halle (Saale), Germany; 15 Friedrich-Schiller-University Jena, Faculty of Biological Sciences Center for Molecular Biomedicine (CMB) Department of Biochemistry Hans-Knöll-Str. 2, 07745 Jena, Germany; 16 Institute for Molecular Virology and Cell Biology (IMVZ), Greifswald, Germany

**Keywords:** Lymphoma, Preclinical research, Apoptosis, Drug development

## Abstract

Histone deacetylases (HDACs) comprise a family of 18 epigenetic modifiers. The biologically relevant functions of HDAC10 in leukemia cells are enigmatic. We demonstrate that human cultured and primary acute B cell/T cell leukemia and lymphoma cells require the catalytic activity of HDAC10 for their survival. In such cells, HDAC10 controls a MYC-dependent transcriptional induction of the DNA polymerase subunit POLD1. Consequently, pharmacological inhibition of HDAC10 causes DNA breaks and an accumulation of poly-ADP-ribose chains. These processes culminate in caspase-dependent apoptosis. PZ48 does not damage resting and proliferating human normal blood cells. The in vivo activity of PZ48 against ALL cells is verified in a *Danio rerio* model. These data reveal a nuclear function for HDAC10. HDAC10 controls the MYC-POLD1 axis to maintain the processivity of DNA replication and genome integrity. This mechanistically defined “HDAC10ness” may be exploited as treatment option for lymphoid malignancies.

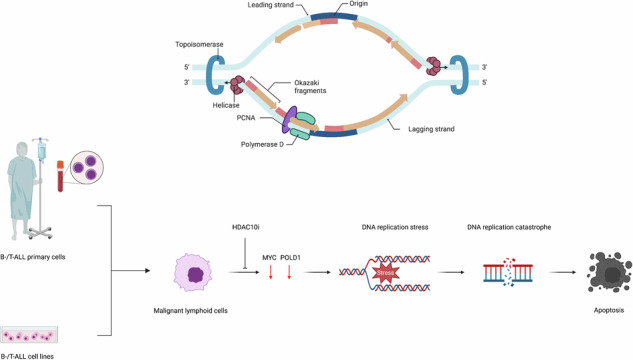

## Introduction

Histone deacetylases (HDACs) and histone acetyltransferases (HATs) control the acetylation of proteins antagonistically [1, 2]. Mammalian HDACs are categorized as class I (HDAC1, HDAC2, HDAC3, HDAC8), class IIa (HDAC4, HDAC5, HDAC7, HDAC9), class IIb (HDAC6, HDAC10), class III (sirtuins SIRT1-7), and class IV (HDAC11) [1, 2].

Epigenetic dysregulation is a key feature of tumorigenesis [3]. Accordingly, tumor and normal cells have different acetylation patterns due to unbalanced levels and activities of HDACs and HATs [1, 2]. Inhibitors of HDACs (HDACi) have been tested in cancer cell models and in the clinic. Four HDACi have been approved by food-and-drug-administration in the US and in China as treatment options for multiple myeloma and cutaneous and peripheral T cell lymphoma. These drugs are the hydroxamic acids vorinostat and belinostat (pan-HDACi), the depsipeptide romidepsin (class I HDACi), and the benzamide chidamide/tucidinostat (inhibitor of class I HDACs and HDAC10). The pharmacological principle of HDACi is a competitive inhibition of the lysine deacetylation reaction. Through polar groups (e. g., hydroxamic acid, thiol moieties) these avidly form complexes with Zn^2+^ in the catalytic pockets of HDACs [4].

Drugs that are pan-HDACi frequently cause therapy-limiting side effects [1, 2, 5, 6]. Studies in cells and mice suggest that isotype-selective HDACi evoke less to no toxic effects on normal cells [7–9]. Unfortunately, there is no in-depth knowledge on which tumor types grow dependently on individual HDACs and accordingly respond best to HDACi. Due to the clinical responsiveness of blood tumors to HDACi [4], leukemia cells are favorable models for such analyses.

HDAC10 exhibits distinctive structural and functional properties. The class IIb HDACs, HDAC6 and HDAC10, have two catalytic domains. However, only one of the catalytic domains of HDAC10 is active [10, 11]. HDAC10 regulates protein homeostasis by modulating the limited process of cellular self-digestion named macroautophagy (hereafter termed autophagy) [10, 12, 13]. Despite its preference for long, slender polyamine substrates in vitro and its mostly cytoplasmic localization, HDAC10 has been reported to deacetylate cytoplasmic and nuclear non-histone proteins. These include cell cycle regulators and proteins mediating DNA synthesis and repair. Consistent with the detectable activity of HDAC10 on histones, a genetic knockout of HDAC10 in non-small cell lung cancer (NSCLC) and melanoma cells augmented the acetylation of histone H3 at K9/K27 and the expression of the cell cycle regulator cyclin A in certain tumor cells [14, 15]. Moreover, HDAC10 deacetylates the DNA mismatch repair protein MutS homolog-2 (MSH2) at K73 [16]. Genetic elimination of HDAC10 sensitizes HeLa cervix carcinoma cells to the DNA-crosslinking agent mitomycin C, by impairing DNA repair through homologous recombination [17]. Furthermore, HDAC10 deacetylates the transcription factor SP1 in NSCLC cells [18].

The role of HDAC10 in leukemia is poorly understood [13]. Artificially overexpressed HDAC10 in cultured chronic lymphoid leukemia (CLL) and mantle B cell lymphoma cells causes cell cycle arrest and apoptosis [19]. Analysis of HDAC expression in 32 primary CLL cells and normal lymphoid cells though revealed that HDAC1, HDAC3, HADC6, HDAC7, HDAC9, HDAC10, SIRT1, and SIRT6 are overexpressed in CLL cells. This is linked to poor prognosis [20, 21]. An association of overexpressed HDAC10 and HDAC7 and reduced HDAC6 and SIRT3 with poor prognosis was also noted when 200 newly diagnosed and relapsed CLL patient samples were compared with normal B cells [22]. Since the constitutive genetic elimination of HDAC10 in mice is not lethal, HDAC10 might be a valid target to treat HDAC10-dependent tumors [7]. It is though unclear whether a pharmacological inhibition of HDAC10 ceases cancer cell growth, how this affects normal cells, and if there are particularly sensitive tumor cell subtypes. Moreover, there are no known molecular biomarkers that can easily and faithfully predict if the effects of an HDAC10 inhibitor are specifically due to HDAC10 inhibition. This contrasts for example, class I HDACi which induce histone hyperacetylation, HDAC6 inhibitors which cause tubulin hyperacetylation, and HDAC8 inhibitors which cause hyperacetylation of structural-maintenance-of-chromosomes-3 (SMC3) [4].

HDAC10 inhibitors can inform about its disease-relevant functions. We and others identified such agents by virtue of the unique negatively charged gatekeeper residue E272 in the catalytic pocket of HDAC10 [23, 24]. These include the hydroxamic acid derivative PZ48 [24]. We used this pharmacological tool, flow cytometry, confocal immunofluorescence, single cell DNA electrophoresis, mRNA-sequencing, mass spectrometry-based proteomics, and quantitative measurement of protein acetylation sites to evaluate the relevance of HDAC10 in human acute B and T lymphocytic leukemia (ALL), acute myeloid leukemia (AML) cells, and lymphoma cells. Our data reveal that human leukemia cells of the lymphoid lineage and lymphomas cells succumb to HDAC10 inhibition, elucidate the underlying molecular mechanisms, and disclose pharmacodynamic markers to assess anti-leukemic activities of HDAC10 inhibitors.

## Material and methods

The full description of the materials and methods that we used to collect our data can be found in the accompanying Supplementary text file *Materials and Methods*. Additional details can be found in the references [25–30].

## Results

### The survival of leukemia cells of the lymphoid lineage depends on HDAC10

**Fig. 1:**
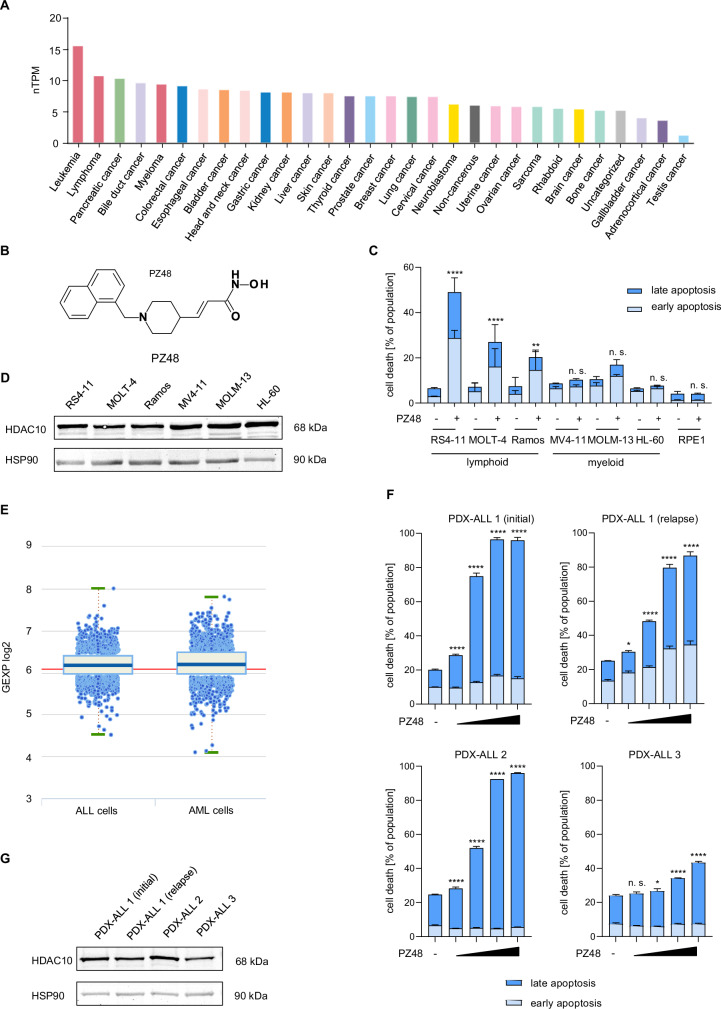
PZ48 is active against lymphoid tumor cells. **A** Comparison of *HDAC10* mRNA expression levels in tumor cell lines, RNA-sequencing data are from 93 leukemia and 76 lymphoma cell lines; database: Cell line - HDAC10 - The Human Protein Atlas. **B** Chemical structure of PZ48. **C** The indicated cell types were treated with 15 µM of PZ48 for 24 h (*n* = 3, mean+SD; two-way ANOVA; ****p ≤ 0.0001). Flow cytometry was used to detect Annexin-V/FITC and PI (-, untreated; +, treated). **D** Immunoblot was done to reveal the expression of HDAC10 in cultured ALL cells; HSP90, loading control. **E** Graphs indicate the expression levels of *HDAC10* mRNA in 1817 ALL cells and 1858 AML cells from patients; GEXP2 log2, logarithmic gene expression; database: http://hemap.uta.fi/. **F** PDX-ALLs were treated with increasing doses (2, 5, 10 and 12 µM; -, control DMSO) of PZ48 for 72 h (*n* = 3, mean+SD; two-way ANOVA; *p ≤ 0.1, ****p ≤ 0.0001). Flow cytometry was used to detect Annexin-V/FITC and PI. **G** Immunoblot shows the levels of HDAC10 in primary ALL cells; HSP90, loading control.

The Human Protein Atlas database [30] illustrates that of various human tumor cell lines analyzed, leukemia and lymphoma cells have the highest *HDAC10* mRNA expression levels **(**Fig. 1A**)**. To clarify if HDAC10 controls leukemia cell fate, we applied the HDAC10 inhibitor PZ48 to human leukemia cells and determined apoptosis by flow cytometry. The chemical structure of PZ48 is depicted in Fig. 1B. The analytical data of PZ48 is included in the Supplementary material and methods. We measured the membrane exposure of phosphatidylserine by staining with Annexin-V and the permeation of cells by propidium iodine (PI). Early apoptotic cells are positive for Annexin-V and late apoptotic cells are positive for Annexin-V and PI [31]. After 24 h, 15 µM PZ48 induced early and late apoptosis significantly in RS4-11 cells (ALL; cells from a 32-year-old female; acute B cell precursor leukemia; 49% apoptosis), in MOLT-4 T lymphoblast-derived cells (from a 19-year-old male at relapse; 27% apoptosis), and in Ramos Burkitt lymphoma cells (from a 3-year-old boy with aggressive non-Hodgkin B cell lymphoma; 20% apoptosis) **(**Fig. 1C**;** *p* = 0.0012-p ≤ 0.0001**)**. However, PZ48 did not induce apoptosis significantly in MV4-11 cells (AML, from a 10-year-old boy), MOLM-13 cells (AML, from a 20-year-old male), HL-60 cells (promyelocytic leukemia, from a 36-year-old female), and RPE1 cells (non-cancerous pigment epithelial cells) **(**Fig. 1C, Supplementary Fig. S1A**)**.

To evaluate if the different responses of AML and ALL cells persist upon longer treatments, we incubated such cells with 5 and 10 µM PZ48 for 72 h. Like in the 24 h treatment schedules, PZ48 induced apoptosis in ALL cells but not in AML cells **(**Supplementary Fig S1B**)**. We can additionally exclude that the different responses of ALL and AML cells to PZ48 can be explained by discrepant HDAC10 protein levels in such cells **(**Fig. 1D**)**. To test these findings in a large set of leukemia cells, we used the online resource Hemap (http://hemap.uta.fi/; [32]). We found that the range of HDAC10 expression levels was similar in 1817 ALL and 1858 AML samples **(**Fig. 1E**)**.

To strengthen the translational relevance of these findings, we incubated primary patient-derived xenograft (PDX) B cell progenitor ALL cells with solvent (DMSO) or 2–12 µM PZ48. We found that 5 µM PZ48 induced 75% apoptosis in KMT2A-rearranged PDX-ALL1 (initial) cells and 48% apoptosis in a corresponding ALL sample from the patient in relapse. Five µM PZ48 induced 52% apoptosis in *TCF3::HLF1*^*+*^ PDX-ALL2 cells. In the ALL sample PDX-ALL3 with hyperactive tyrosine kinase signaling (*BCR::ABL1*^*+*^) 12 µM PZ48 evoked 43% apoptosis **(**Fig. 1F; *p* ≤ 0.0001**)**. Immunoblotting illustrates that these cells express HDAC10 **(**Fig. 1G**)**.

These findings reveal that a subset of cultured and primary leukemic cells relies on HDAC10 for their survival. The underlying mechanism is more complex than different responses due to divergent HDAC10 expression levels.

### Analysis of the specificity of PZ48 for HDAC10

We aimed to corroborate that specifically HDAC10 is necessary for the survival of RS4-11 cells which are the most PZ48-sensitive cells in our panel. As cell system for comparison, we used MV4-11 cells. These express HDAC10 but are not killed by PZ48 **(**Fig. 1C, Supplementary Fig. S1B**)**. We also chose these two cell systems because both carry oncogenic translocations in the mixed-lineage leukemia gene. The available HDACi can disclose individual HDAC functions [4]. We used MS-275 to inhibit HDAC1,-2,-3, Marbostat-100 to selectively inhibit HDAC6, TMP269 to inhibit HDAC4,-5,-7,-9, and the HDAC8 inhibitors PS23 and PCI34051. To verify the specific targeting of individual HDACs or their classes, we measured the acetylation of histone H3 at the N-terminus as target of class I HDACs, the acetylation of tubulin being the prototypical target of HDAC6, and the HDAC8-regulated acetylation of SMC3 [4]. PZ48 did not augment the acetylation of these proteins **(**Supplementary Fig. S1C-D**)**.

We could support these findings with tests for apoptosis induction. PZ48 is more effective against RS4-11 cells than MV4-11 cells **(**Fig. 1C, Supplementary Fig. S1B**)**. If PZ48 blocks class I HDACs or class IIa HDACs, RS4-11 cells will also be more sensitive to inhibitors of these enzymes. Unlike PZ48, both MS-275 and TMP269 induced higher levels of apoptosis in MV4-11 cells than in RS4-11 cells **(**Supplementary Fig. S1E-F**)**. HDAC8 inhibitors did not harm RS4-11 cells **(**Supplementary Fig. S1G**)**. These datasets suggest that PZ48 does not have relevant non-specific effects on these HDACs. HDAC6 and HDAC10 are the two class IIb HDAC members. Since we previously found that RS4-11 cells and MV4-11 cells are not killed upon a specific HDAC6 inhibition [33], we can rule out that an impact of PZ48 on HDAC6 induced apoptosis in RS4-11 cells.

A molecular marker for HDAC10 inhibition is dysregulated autophagy [34]. We previously showed that PZ48 modulated the autophagosomal-lysosomal compartment in MV4-11 cells [24]. Therefore, we evaluated if a differential modulation of autophagy by PZ48 in MV4-11 and RS4-11 cells could explain their differential responses to PZ48. We analyzed the accumulation of autophagosomes in RS4-11 and MV4-11 cells using CYTO-ID staining. PZ48 induced an accumulation of autophagosomes in both cell lines upon co-treatment with chloroquine that inhibits the fusion of autophagosomes and lysosomes **(**Supplementary Fig. S1H**)**. These data disfavor that MV4-11 cells do not take up PZ48 or export PZ48 via multidrug resistance pumps. Moreover, we could verify that PZ48 induced apoptosis in RS4-11 cells using the pan-caspase inhibitor Z-VAD-FMK **(**Supplementary Fig. S1I**)**.

When we compared the inhibitory profiles of PZ48 and six pan- or isoform-specific HDACi in an in vitro assay with ten purified HDACs, PZ48 turned out as a low nanomolar inhibitor of HDAC10 **(**Table 1; 2.7 ± 0.3 nM**)**. In this assay, PZ48 inhibits HDAC8 179-fold less effectively. The finding that HDAC8 inhibitors do not induce apoptosis of RS4-11 cells (Supplementary Fig. S1G) preclude that PZ48 kills such cells through inhibition of HDAC8.

**Table 1:** In vitro data for PZ48; the table highlights data for PZ48 and how the tested HDACi affect HDAC10; empty fields stand for tests that were not done; HDAC, histone deacetylase; IC50, half-maximal inhibitory concentration.

Name	IC50 HDAC1 [nM]	IC50 HDAC3 [nM]	IC50 HDAC4 [nM]	IC50 HDAC5 [nM]	IC50 HDAC6 [nM]	IC50 HDAC7 [nM]	IC50 HDAC8 [nM]	IC50 HDAC9 [nM]	HDAC10 IC50 [nM]	HDAC11 IC50 [nM]
**PZ48**	**3 000** ± **200**	**>20 000**	**3 000** ± **560**	**1 010** ± **200**	**3 700** ± **450**	**940** ± **70**	**465** ± **73**	**2 200** ± **390**	**2.66** ± **0.29**	**>20 000**
**Vorinostat**	101 ± 7	210 ± 10			42 ± 11		400 ± 100			
**Entinostat (MS-275)**	940 ± 130	1800 ± 100	>20 000	>20 000	>20 000	>20 000	>20 000	>20 000	**>20 000**	>20 000
**Nexturastat**					35 ± 5		410 ± 33			
**PC34501**	>20 000	>20 000			>20 000		92 ± 15		**>20 000**	
**TMP269**	>20 000		640 ± 150	570 ± 150		43 ± 5	>20 000	37 ± 3	**>20 000**	
**SIS17**	>20 000	>20 000	>20 000	>20 000	>20 000	>20 000	>20 000	>20 000	**>20 000**	170 ± 20

These data illustrate that a non-selective inactivation of HDACs by PZ48 should not explain its pro-apoptotic effects towards ALL cells.

### Inhibition of HDAC10 reduces the expression of POLD1

**Fig. 2:**
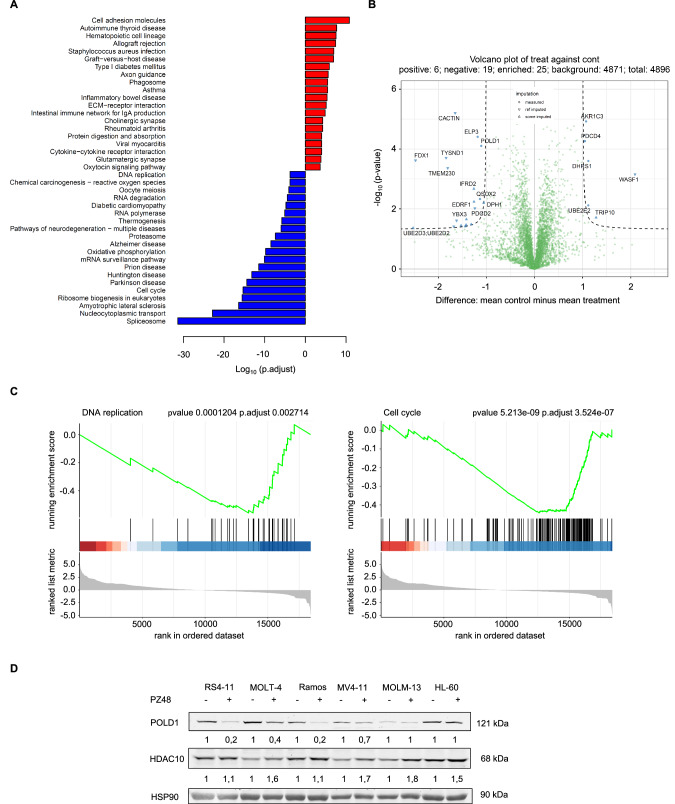
Inhibition of HDAC10 alters key cellular processes and downregulates POLD1. **A** The top 20 KEGG pathways enriched in the list of up-regulated (red) and down-regulated (blue) genes in the PZ48-treated samples with an FDR < 0.05. **B** Volcano blot shows differently expressed proteins of RS4-11 cells that remained untreated or were treated with 15 µM PZ48 for 24 h. **C** GSEA plots show the DNA replication gene set (left) or the cell cycle gene set (right) enriched in the down-regulated genes in PZ48-treated RS4-11 cells with an FDR < 0.05. **D** RS4-11, MOLT-4, Ramos, MV4-11, MOLM-13, and HL-60 cells were treated with 15 µM PZ48 for 24 h (+; -, untreated). Immunoblot analyses were done as indicated (*n* = 3).

To unravel the mechanisms underlying apoptosis induction upon HDAC10 inhibition with PZ48, we performed an RNA sequencing analysis on RS4-11 cell samples. We limited treatment times with PZ48 to 8 h to avoid an overrepresentation of apoptosis-associated events and of processes that follow cell cycle alterations **(**Supplementary Fig. S2A, B**)**. Among the altered gene expression profiles upon HDAC10 inhibition were genes that control key biological processes including DNA replication, upstream regulators of the cell cycle, and hematopoietic identity. As expected, apoptotic signatures were not prevalent **(**Fig. 2A**)**.

To evaluate which of these differences in transcriptional output translate into changes in the proteome of PZ48-treated RS4-11 cells, we performed mass spectrometry-based proteomics. We found that the catalytic subunit of the DNA-polymerase δ, POLD1, was among the altered proteins **(**Fig. 2B**)**. The DNA polymerase δ complex, which synthesizes the lagging DNA-strand during DNA synthesis in S phase, contains the catalytic subunit POLD1 and the accessory subunits POLD2 and POLD3 [35].

These findings correspond to gene ontology (GO) terms in our transcriptomic data **(**Fig. 2A**)**. Consistent with the proteomics data shown in Fig. 2B and the key role of POLD1 for DNA synthesis, gene set enrichment analyses (GSEA) illustrate a strongly altered regulation of genes being required for cell cycle progression and DNA replication **(**Fig. 2C**)**. Analyzing the database DepMap, we noted a significant correlation (Pearson *r* = 0,781; Spearman ρ = 0,671; *p* = 0.000004) between the expression of HDAC10 and POLD1 in 32 human lymphoblastic leukemia and lymphoma cell lines **(**Supplementary Fig. S2C**)**.

The data above suggests the working hypothesis that HDAC10 maintains the expression of POLD1 in cells that undergo apoptosis in the presence of PZ48. To scrutinize the interplay between HDAC10 and POLD1 expression, we assessed their levels by immunoblot in various leukemia cell types that were incubated with PZ48. PZ48 decreased POLD1 in RS4-11, MOLT-4, and Ramos cells, but not significantly in MV4-11, MOLM-13, and HL-60 cells **(**Fig. 2D**)**. This downregulation of POLD1 correlates with the pro-apoptotic effects of PZ48 **(**Fig. 1C, Supplementary Fig. S1B**)**. PZ48 did slightly induce an accumulation of HDAC10 in all these cell lines **(**Fig. 2D**)**. Therefore, we can exclude that a disruption of HDAC10 expression reasons the sensitivity of RS4-11, Ramos, and MOLT-4 cells to pro-apoptotic effects of PZ48 **(**Fig. 1C, Supplementary Fig. S1A, B**)**.

These results disclose that HDAC10 controls the POLD complex, which is an indispensable regulator of DNA replication

### Impairment of HDAC10 causes DNA damage promoting apoptosis

**Fig. 3:**
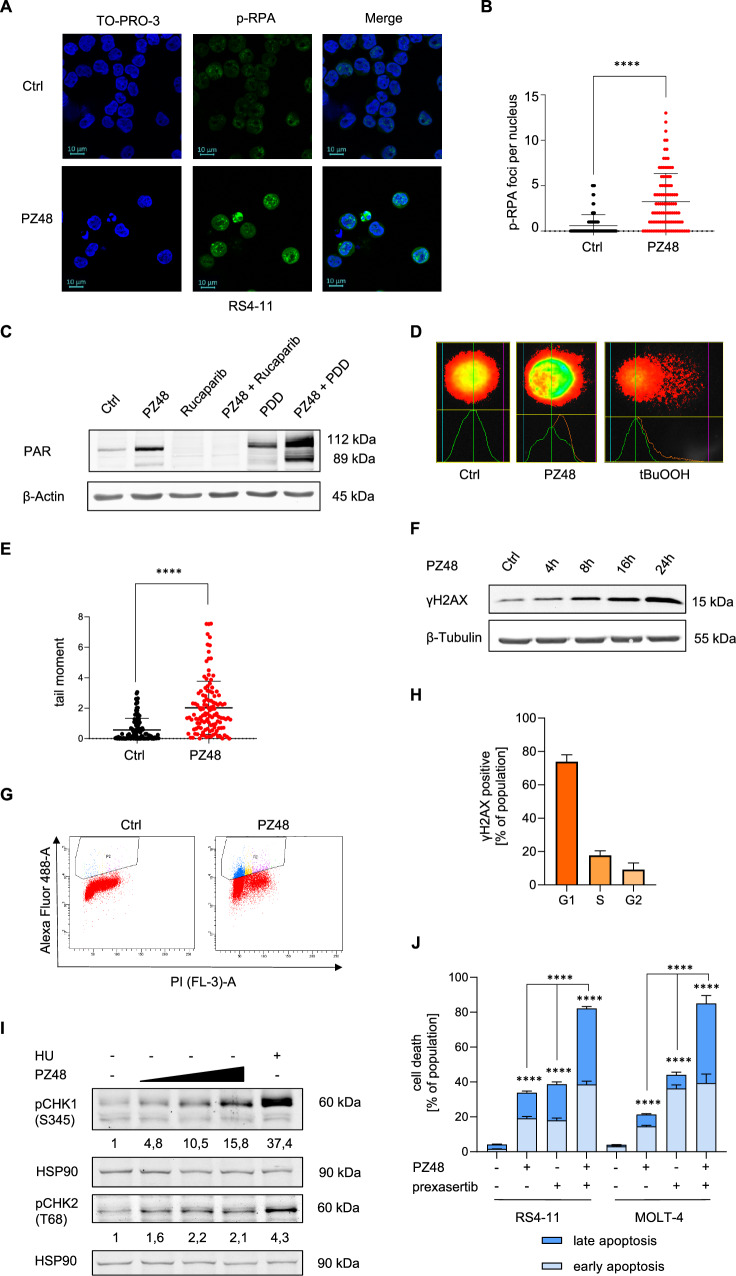
PZ48 induces DNA replication stress and DNA damage. **A** RS4-11 cells were incubated for 24 h with 10 µM PZ48 (lower) or remained untreated (upper, *n* = 3). Immunofluorescence staining was done to detect phosphorylated RPA (p-RPA). TO-PRO-3 was used to stain nuclei. The scale bars represent 10 µm. **B** Quantification of **A** showing p-RPA foci per nucleus. 100 nuclei were counted (Mann-Whitney U; **** *p* ≤ 0.0001). **C** Immunoblot analysis detected PAR chains in lysates from RS4-11 cells that were treated with 15 µM PZ48, 10 µM Rucaparib or 2 µM PDD00017273 (PDD) alone or in combination. HSP90 serves as a loading control (*n* = 3). **D** Representative pictures of alkaline single cell gel electrophoresis assays in RS4-11 cells. Comparison of cells used as untreated controls (left), 4 h treatment with 15 µM PZ48 samples (middle), and positive control cells for DNA replication stress/DNA damage (4 h treatment with 200 µM tBuOOH, right). **E** Quantification of **D**, 100 cells were counted per slide (Mann-Whitney U; **** *p* ≤ 0.0001; *n* = 3). **F** Immunoblot analysis detected phosphorylated H2AX (γH2AX) in lysates of RS4-11 cells. These were treated with 15 µM PZ48 for the indicated time periods. β-tubulin serves as loading control (*n* = 3). **G** Shown is an example of PI/Alexa fluor 488 (γH2AX) staining of RS4-11 cells. These were treated for 8 h with 15 µM PZ48 and analyzed by flow cytometry. Cells above the population of untreated cells are considered as being γH2AX-positive. **H** Quantification of γH2AX-positive cells for each cell cycle phase (*n* = 3). **I** Immunoblot analysis was used to detect phosphorylated forms of CHK1 and CHK2 in RS4-11 cell lysates. The cells were treated with increasing doses of PZ48 (5 µM, 10 µM, 15 µM) in comparison with untreated control cells and cells treated with 1 mM hydroxyurea as positive control for CHK activation. HSP90 serves as loading control (*n* = 3). **J** RS4-11 and MOLT-4 cells were incubated with 15 µM PZ48 ± 10 nM prexasertib for 24 h. The cells were processed for flow cytometry measuring early and late apoptosis. Data are mean values from 3 independent experiments (mean+SD; two-way ANOVA; ****p ≤ 0.0001).

We used confocal immunofluorescence to investigate whether the reduction of POLD1 by PZ48 and the subsequent cell cycle delay were linked to DNA replication stress and DNA damage. Nuclear foci containing the phosphorylated DNA replication protein-A (RPA; ssDNA-binding protein) indicate ssDNA stretches in the genome [36]. The treatment of RS4-11 cells with 10 µM PZ48 for 24 h increased p-RPA foci significantly **(**Fig. 3A, B**)**.

The enzyme poly(ADP-ribose)-polymerase 1 (PARP1) is activated immediately upon DNA replication stress and can promote apoptosis and other cell death modes [37]. Poly-ADP-ribose (PAR) chains are transferred to target proteins upon DNA replication stress by PARP1. Hyperactivation of PARP1 and accumulation of PAR indicate DNA damage [38]. Immunoblot analyses showed a strong autoPARylation of PARP1 upon treatment with PZ48 **(**Fig. 3C**)**. The compound PDD00017273 inhibits PARG which detaches PAR [38]. Consistently, the accumulation of PAR was more detectable when PDD00017273 was given with PZ48 and the selective PARP1 inhibitor rucaparib prevented PZ48-induced PARylation in RS4-11 cells **(**Fig. 3C**)**.

The alkaline single cell DNA assay, which is also known as comet assay, detects ssDNA lesions and DNA double strand breaks (DSBs) in individual cell nuclei [39]. This approach showed that PZ48 induced DNA lesions in RS4-11 cells significantly **(**Fig. 3D, E**)**.

Immunoblotting for the DNA replication stress/damage marker phosphorylated histone H2AX (ɣH2AX) [36] confirmed the accumulation of DNA lesions in PZ48-treated RS4-11 cells **(**Fig. 3F**)**. To assess in which cell cycle phase such lesions occur, we used a flow cytometry-based co-detection of ɣH2AX and DNA content. We found an increase in ɣH2AX-positive RS4-11 cells in the early G1 phase/S phase traversal **(**Fig. 3G, H), i.e., the cell cycle phase that determines entry into S phase.

Checkpoint kinases (CHKs) orchestrate cell cycle arrest upon stalled DNA replication and DNA damage [36, 40]. Immunoblot analyses showed that the compromised DNA integrity in PZ48-treated cells was linked to the phosphorylation-dependent activation of CHK1 and CHK2 in RS4-11 cells. This effect of PZ48 on CHK1 occurred in a dose-dependent manner **(**Fig. 3I**)**, reflecting the concentration-dependent apoptosis induction by PZ48 **(**Supplementary Fig. S1B**)**.

Overactivation of CHK1 and CHK2 can promote the survival of stressed cancer cells by allowing them to temporarily halt the cell cycle for repair, highlighting the potential for combination treatments with inhibitors of CHK1 and CHK2 [40]. We treated RS4-11 cells and MOLT-4 cells with PZ48 and the clinically tested CHK1 inhibitor prexasertib or PZ48 and the CHK2 inhibitor II hydrate. We found that PZ48 combined favorably with prexasertib against these leukemia cells. These effects were significant **(**Fig. 3J**)**. The CHK2 inhibitor did not augment the PZ48-induced apoptosis **(**Supplementary Fig. S3A**)**.

To assess if PZ48-induced DNA damage is mutagenic, we used a bacterial reverse mutation test using the *Salmonella typhimurium* strain TA100. There were no revertant colonies visible in DMSO-treated control samples and when using up to 15 µM PZ48 after 48 h. The positive control of 0,5 mg sodium azide caused over 700 reverse mutations **(**Supplementary Fig. S3B, S3C**)**. These findings suggest that PZ48 is non-mutagenic.

These results show that PZ48 induces DNA lesions in leukemia cells that undergo apoptosis in response to this drug. This DNA replication stress induction creates a vulnerability to CHK1 inhibitors.

### PZ48 controls MYC-POLD1 signaling

**Fig. 4:**
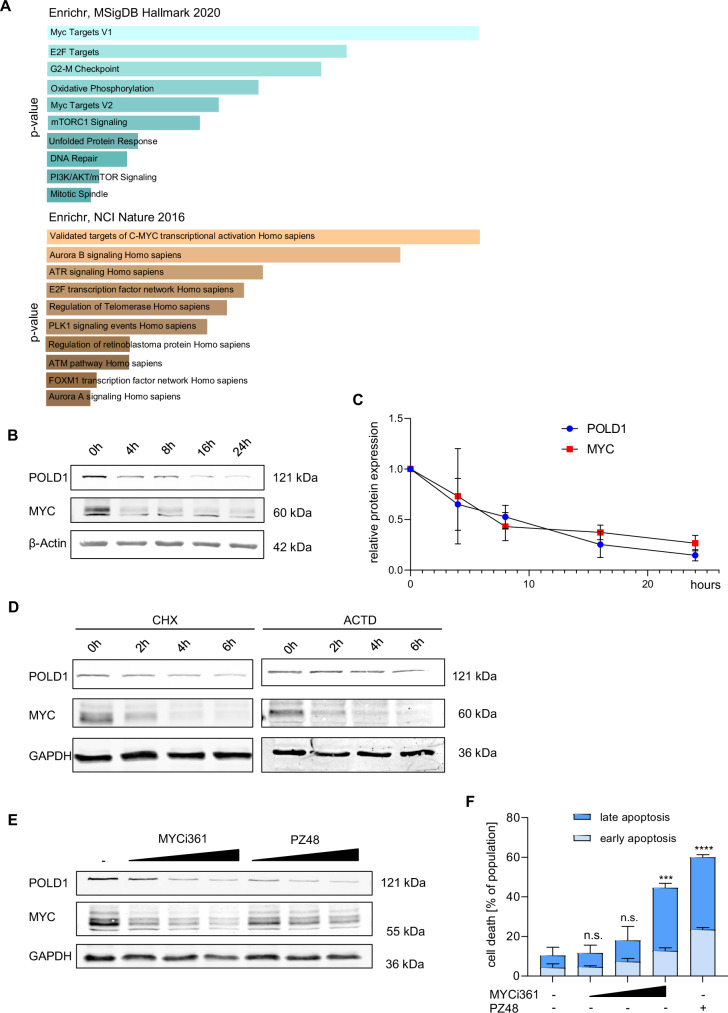
MYC is lost rapidly upon the inhibition of HDAC10. **A** Pathway analysis of RNA sequencing data of RS4-11 cells treated with 15 µM PZ48 for 8 h (see Fig. 2A) using the Enrichr database. Shown are the MSigDB Hallmark 2020 data set (upper) and the NCI Nature 2016 data set (lower). Genes that we found downregulated in RNA sequencing analyses were used as input and an FDR < 0.05 was set; *p*-values increase from top to bottom. **B** Immunoblot analysis of RS4-11 cells that were treated with 15 µM PZ48 for increasing time periods (4 h, 8 h, 16 h, 24 h, *n* = 3). β-actin serves as loading control. **C** Quantification of **B** showing the mean ± SD values of 3 independent experiments. **D** RS4-11 cells were exposed to cycloheximide (CHX, left, 20 µM) and actinomycin D (ACTD, right, 2 µg/ml) for 2 h, 4 h, and 6 h. Immunoblot was done for POLD1 and MYC; GAPDH serves as loading control (*n* = 3). **E** Immunoblot was conducted with lysates from RS4-11 cells that were treated with increasing doses of MYCi361 (5 µM, 10 µM, 15 µM) in comparison to PZ48 (5 µM, 10 µM, 15 µM) for 24 h. GAPDH serves as loading control (*n* = 3). **F** RS4-11 cells were treated with MYCi361 (5 µM, 10 µM, 15 µM) for 24 h in comparison to untreated negative control cells (-) and treatment with 15 µM PZ48 (+) as positive control for apoptosis induction. Flow cytometry was used to detect Annexin-V/FITC and PI as early/late apoptosis markers (*n* = 3, mean+SD; two-way ANOVA; ****p ≤ 0.0001).

Having assessed that HDAC10 controls POLD1 expression, we asked which transcription factor(s) controlled this newly discovered mechanism. Pathway analyses of our RNA-sequencing data revealed targets of the transcription factor MYC as top hits in RS4-11 cells that were treated with PZ48 **(**Fig. 4A**)**. MYC controls cell cycle regulation, DNA replication, and apoptosis [41]. PZ48 affects these processes **(**Figs. 1–3**)**. RNA-sequencing analysis consistently demonstrated that PZ48 decreased MYC expression **(**Supplementary Fig. S4A**)** and the expression of genes encoding MYC-regulated polymerase subunits **(**Supplementary Fig. S4B**)**.

To evaluate if a reduction of MYC is associated with a decrease of POLD1 in PZ48-treated cells, we incubated RS4-11 cells with PZ48 for 4-24 h and did quantitative immunoblot analyses. We found that a loss of MYC tied in with the attenuation of POLD1 **(**Fig. 4B**)**. Both effects were detectable after 4 h, with over 50% reduced levels of MYC and POLD1 after 8 h **(**Fig. 4C**)**.

To determine if the loss of MYC and POLD1 is linked to changes in their acetylation levels, we used stable isotope labeling with amino acids in cell culture (SILAC)-based quantitative mass spectrometry. We compared the acetylation levels of proteins in untreated and PZ48-treated RS4-11 cells. We quantified differently acetylated sites within 127 proteins. Intriguingly, MYC was the only protein shown to be significantly deacetylated, which likely reflects its decreased expression. Matching the protein deacetylase function of HDAC10, many proteins, including histones, were shown to be hyperacetylated in the presence of PZ48. POLD1 was not found to be an acetylated protein **(**Supplementary Fig. S4C**)**.

A rapid reduction of MYC in PZ48-treated RS4-11 cells after 2 h disfavors that this processes is a consequence of cell cycle alterations or apoptosis **(**Supplementary Fig. S4D**)**. We could corroborate this conclusion with a combinatorial treatment with PZ48 and Z-VAD-FMK. Although Z-VAD-FMK prevented the cleavage of caspase-3, it did not restore MYC or POLD1 expression levels **(**Supplementary Fig. S4E**)**. This data and our observation of no significant apoptosis occurring after a 2 h-treatment with PZ48 **(**Supplementary Fig. S4F**)** excludes apoptotic protein degradation as an explanation for the breakdown of the MYC-POLD1 axis upon HDAC10 inhibition.

If the PZ48-evoked, rapid loss of POLD1 is a consequence of disrupted MYC-dependent gene expression, an acute shutdown of protein translation or mRNA transcription will reduce MYC and POLD1. Incubation of RS4-11 cells with the translation inhibitor cycloheximide (CHX) or the RNA polymerase inhibitor actinomycin-D (ACTD) attenuated MYC levels after 2 h. This was followed by a gradual reduction of POLD1 **(**Fig. 4D**)**.

Using the MYC inhibitor MYCi361, we assessed its anticipated functional relevance on the MYC-POLD1 axis. MYCi361 phenocopied the reduction of protein levels of POLD1 and MYC in PZ48-treated RS4-11 cells **(**Fig. 4E**)**. Assessing apoptosis induction by flow cytometry, we could verify that MYC is a survival factor in such cells **(**Fig. 4F**)**.

These data support our hypothesis that the inhibition of HDAC10 in lymphoid cells decreases MYC expression, resulting in reduced expression of POLD1, and ultimately cell cycle arrest and parthanatos-associated apoptosis.

### PZ48 eliminates ALL cells in vivo within a therapeutic window sparing normal cells

**Fig. 5:**
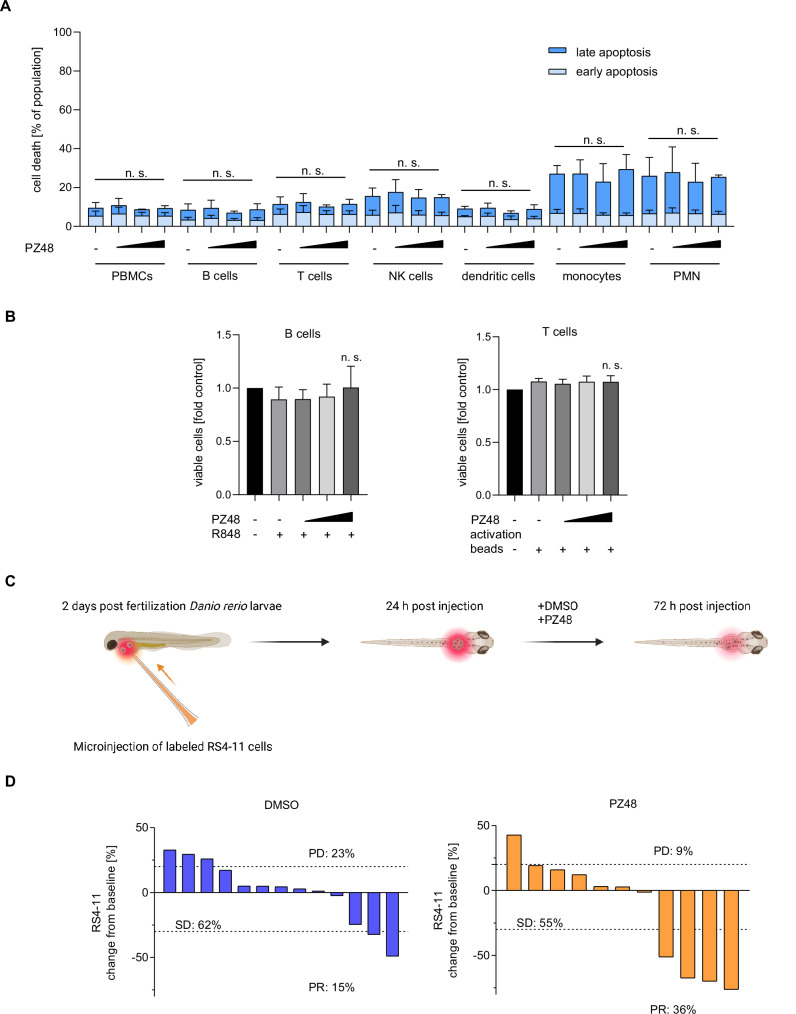
PZ48 does not harm normal blood cells and is effective against ALL cells in vivo. **A** PBMCs were treated with increasing doses (5 µM, 10 µM, 15 µM) of PZ48 for 24 h. Staining of cells was done with Annexin-V AF647 and FVD eFl780. The cells were analyzed using flow cytometry (*n* = 3, mean+SD; two-way ANOVA; n.s., not significant). Isolated subtypes of cells were defined: CD3-CD19+ as B cells; CD3+ as T cells; CD3-CD19-CD56+ as NK cells; CD3-CD19-CD1c+ as dendritic cells; CD3-CD19-CD14+ as monocytes; and CD3-CD14-CD19-CD56-CD11b+ as PMNs. **B** PBMCs were incubated with R848 (1 µg/ml) or Dynabeads™ Human T-Activator CD3/CD28 (5 µl/ml) for 24 h (control, DMSO treatment). Then, PBMCs were treated with increasing doses (5 µM, 10 µM, 15 µM) of PZ48 for 24 h. Staining of FVD eFl780 was analyzed using flow cytometry (*n* = 5, mean+SD; two-way ANOVA; n.s., not significant). T cells were defined as CD3 + , B cells were defined as CD3-CD19 + . **C** Model of the *Danio rerio* experiment. Created in BioRender. Mieland, A. (2025) https://BioRender.com/t85m878. **D** Waterfall plots demonstrating changes in tumor volume [%] for each individual zebrafish larvae engrafted with RS4-11 cells, from baseline (day 1 = start of the treatment) until day 3 after injection of such cells. Zebrafish larvae xenografts were treated with DMSO used as a solvent (*n* = 13 larvae; left) or 40 µM PZ48 (*n* = 11 larvae; right) for 48 h; each bar reflects one individual xenograft. Numbers indicate the percentages of early larvae with progressive disease (PD), stable disease (SD), and partial response (PR) in each treatment group on day 3.

To evaluate if the cytotoxic effect of PZ48 was restricted to lymphoid tumor cells, we used human peripheral blood mononuclear cells (PBMCs) from healthy donors. Since PBMCs consist of cells of the lymphoid and myeloid lineages, we discriminated individual cell types by flow cytometry using lineage-specific markers. We found that 5–15 µM PZ48 did not augment apoptosis in any of these cell populations **(**Fig. 5A**)**.

Next, we studied whether PZ48 affected proliferating B and T cells. We chose these cell types because PZ48 kills B and T lymphoblastic leukemia cells **(**Fig. 1C, Supplementary Fig. S1B**)**. We stimulated B cell proliferation with the toll-like receptor agonist resiquimod (R848) and induced T cell proliferation by T cell receptor activation beads. The treatment of such cells with 5-15 µM PZ48 did not compromise their viability **(**Fig. 5B**)**.

We then analyzed if normal hematopoietic stem and progenitor cells (enriched in the stem cell marker CD34) from healthy donors maintain colony-forming capacity ex vivo in the presence of 5 µM PZ48 **(**Supplementary Fig. S5A**)**. PZ48 treatment resulted in a minor reduction of colony numbers compared to solvent-treated cells. Over 80% of colonies were formed **(**Supplementary Fig. S5B**)**. Analogous experiments with RS4-11 cells in methylcellulose demonstrated that such cells were more affected than normal CD34-enriched cells by PZ48. This particularly affected the size of RS4-11 cells colonies and the respective cell counts **(**Supplementary Fig. S5C**)**.

These findings encouraged us to assess the efficacy of PZ48 in vivo. We first conducted a toxicity assay with 5–40 µM PZ48 that we applied to *Danio rerio* larvae. No signs of toxicity were observed using up to 40 µM of PZ48 **(**Supplementary Fig. S5D**)**. We then injected RS4-11 cells into early *Danio rerio* larvae, allowed the cells to establish leukemia cell masses, and then treated them with PZ48 or its solvent DMSO for 48 h **(**Fig. 5C**)**. Larvae with at least 20% increase or 30% decrease in tumor volume were classified as having progressive disease (PD) or partial response (PR). Other larvae were considered to have stable disease (SD). In the solvent-treated group, 62% of larvae had SD, 23% had PD, and 15% had PR. The response rate of xenografts upon treatment with PZ48 became 55% SD and PR increased more than two-fold to 36%. PD was reduced to 9% **(**Fig. 5D**)**.

These data show that PZ48 halts the proliferation of ALL cells in a bone marrow-resembling matrix and in vivo, and that PZ48 has low to non-significant toxic effects on normal cells and tissues.

## Discussion

Our results illustrate that a pharmacological inhibitor of HDAC10 evokes apoptosis specifically in lymphoid tumor cells. ALL is difficult to treat in adults. Although this leukemia is better treatable in children, the required chemotherapy is associated with high co-morbidity and lasting tissue and organ damage [42–45]. We noted that PZ48 caused no acute damage when applied to normal human PBMCs and PZ48 did not impair the growth of activated B and T lymphocytes. The survival of normal cells in the presence of an HDAC10 inhibitor reflects the phenotype of HDAC10 null mice [7, 9]. Further studies will show if such inhibitors are innovative and safe anti-cancer drugs. The frequently found overexpression of more than one HDAC in leukemia cells suggests that broader acting HDACi may be more useful than selective ones. However, overexpression does not undisputably mean overactivation. For example, the intracellular localization, posttranslational modifications, and the expression of specific targets of HDACs can determine their functional relevance in cells.

Several studies analyzed whether treatment with HDACi achieves clinical benefits. Since food-and-drug-administrations approved four HDACi for the treatment of cutaneous and peripheral T cell lymphoma, HDACi may also be useful to treat ALL patients [46]. A recent study evaluated chidamide as an additional treatment for chemotherapy or after hematopoietic stem cell transplantation in 27 children (mean age 7.9 years, male:female = 8:1, median follow-up period 37.8 months) with T-ALL. Of these, 25 remained in remission (<0.01% blast cells) [46]. In a trial that assessed the pan-HDACi vorinostat plus the DNA methyltransferase inhibitor decitabine with chemotherapy in 23 children (mean age 12 years), 15 had a complete response or stable disease. Despite good pharmacokinetic data, a high incidence of infectious toxicities led to a cessation of the study [47]. This perhaps indicates that the more selective HDACi chidamide is more suitable than the pan-HDACi vorinostat. Two case reports involving one 46-year-old adult with relapsed B-ALL harboring a mixed-lineage leukemia fusion gene and two adult male patients with T-ALL carrying the SET-NUP214 fusion present promising data on a safe use of chidamide with the BCL2 protein inhibitor venetoclax and azacytidine or post-hematopoietic stem cell transplantation [48, 49]. Obviously, more clinical data are required to judge the usefulness of HDACi in patient settings. This holds for other hematologic malignancies. For example, in a phase Ia/II study, single-agent activity of the pan-HDACi panobinostat was observed in Hodgkin lymphoma and myelofibrosis [50]. Thus, it is possible that PZ48 is useful to treat such diseases.

Undisputedly, specific HDAC10 inhibitors are tools to elucidate the largely unknown targets and biological functions of HDAC10. Our data show that a specific inhibition of HDAC10 kills a subset of leukemic cell types with lymphatic origin. A molecular characteristic of such cells is that they require HDAC10 to maintain the expression of MYC and POLD1 (i.e., HDAC10ness in analogy to the BRCAness of certain tumor cells). Such a MYC-POLD1 signaling axis also occurs in bladder cancer [51], suggesting that further tumor cell types could be susceptible to HDAC10 inhibitors. Further studies are needed to clarify why certain leukemia cells are resistant to PZ48. We speculate that hyperactive kinases, such as FLT3-ITD in AML cells or BCR-ABL in chronic myeloid leukemia cells and a subset of ALL cells [52], activate MYC and POLD1, and that this attenuates the cytotoxic effects of HDAC10 inhibition.

Analysis of the acetylome revealed that PZ48 induced a hyperacetylation of histones. The acetylation status of histones plays a pivotal role in controlling transcription in cells, confirming that HDAC10 controls epigenetic mechanisms [15]. Exploiting such tumor-associated functions of HDAC10 requires the identification of biomarkers for the anticancer activity of its inhibitors. We show that the loss of the MYC-POLD1 axis and the resulting DNA replication stress response indicate anti-leukemic drug efficacy. The induction of autophagy seems unsuitable as such a marker, as it is induced upon HDAC10 inhibition irrespective of apoptosis.

Hydroxamic acids were reported to be attached to DNA and to consequently cause DNA damage [53]. As we do not observe such general DNA damage, we conclude that the DNA replication stress/DNA damage phenotype that we see is linked to and specific for leukemia cells that succumb to apoptosis in response to PZ48. The depletion of POLD1 by PZ48 appears to be sufficient to trigger DNA replication stress in such cells.

The PZ48-induced DNA replication problems trigger an activation of checkpoint kinases that suppress cell cycle progression and induce DNA repair processes. If the DNA damage is too severe, checkpoint kinases activate apoptosis inducers including the transcription factors p53 and p73 [54]. We found that PZ48 combined favorably with the CHK1 inhibitor prexasertib against ALL cells. Such a finding is coherent with our notion that PZ48 depletes POLD1 and consequently causes DNA replication fork stalling. This decrease in lagging DNA strand synthesis, that is carried out by POLD1, leads to single-stranded DNA stretches and single strand DNA breaks. These trigger the activation of the ATR-CHK1 axis, for which we identify CHK1 as susceptibility factor in PZ48-treated ALL cells. A CHK2 inhibitor did not augment the PZ48-induced apoptosis. This corresponds to the notion that the ATM-CHK2 axis is activated after double strand DNA breaks [40]. Experiments are underway to address the biological relevance of PARP1 activation and the associated parthanatos accompanying apoptosis. It will also be interesting to delineate how inhibition of HDAC10 interacts with inhibitors of cell cycle regulatory kinases, such as the WEE1 inhibitor adavosertib which disables inhibitory phosphorylation of CDK1/CDK2 and proper DNA synthesis [55].

The replicative DNA polymerases Polα/POLA, Polδ/POLD, and Polɛ/POLE are necessary for DNA replication in the S phase and promote DNA repair and recombination processes. The DNA-POLD complex is indispensable for DNA synthesis and additionally contributes to DNA repair mechanisms like nucleotide excision repair or mismatch repair [35]. Thus, PZ48 might equally be combined favorably with chemotherapeutics that cause DNA base pair mismatches and bulky DNA adducts in leukemia cells.

In conclusion, we unravel how HDAC10 affects human leukemic cells. We show that an inhibitor of HDAC10 ceases a MYC-POLD1 axis and consequently causes DNA replication stress, DNA damage, parthanatos, and apoptosis in B and T ALL cells. Despite such profound effects, normal cells are not significantly damaged upon pharmacological HDAC10 inhibition. We prove the efficacy of the HDAC10 inhibitor PZ48 in a *Danio rerio* larvae model and speculate that HDAC10 inhibitors might be prospectively used to treat aggressive lymphocytic leukemia.

## Supplementary information

Mieland et al._Material&Methods SI

Figs. S1-S5

Mieland et al Original blots

## Data Availability

RNA-seq data generated and analyzed in this study are available in the Gene Expression Omnibus repository, https://www.ncbi.nlm.nih.gov/geo, under accession number GEO: GSE277944. To review the data while it remains in private status: https://www.ncbi.nlm.nih.gov/geo/query/acc.cgi?acc=GSE277944 and use the token yxcbkesqnpctxob. Mass spectrometry raw data have been deposited to the ProteomeXchange Consortium via the PRIDE partner repository with the dataset identifiers PXD056251 and PXD058544.
