# Supplementary material for: The protein deacetylase HDAC10 controls DNA replication in malignant lymphoid cells
Source: Leukemia. 2025 Apr 29;39(7):1756–68. doi: 10.1038/s41375-025-02612-8 (PMC12208866; doi:10.1038/s41375-025-02612-8)
Supplement: Supplementary file 1 — Mieland et al._Material&Methods SI [file 41375_2025_2612_MOESM1_ESM.docx]

**Supplementary material and methods**

**HDAC in vitro assay**

Human HDAC10 full-length protein was expressed and purified as described (1). A fluorescence based HDAC10 enzymatic assay was used (2). For the fluorescence measurements, a PerkinElmer Envision 2104 multilabel plate reader (Waltham, MA, USA) was used at λex = 320 nm and λem = 430 nm. The reaction mixture consisted of HDAC10, and the acylated peptide substrate derived from TNFα in a reaction buffer comprising 50 mM HEPES, 2 mg/mL BSA, and 70 µMTCEP, and at pH 7.4 which was adjusted with NaOH (total volume 40 μL) (2). The reactions were incubated in black 384-well plates for 30 min (scan every 30 s) at room temperature, and the increase of relative fluorescence reflecting the product formation was monitored. As reference for HDAC11 inhibitors we used the reported compound SIS17 (purchased from MedChemExpress LLC, 1 Deer Park Dr, Suite Q, Monmouth Junction, NJ 08852, USA).

For HDAC1, 2, 3 and HDAC6 the recombinant proteins were purchased from ENZO Life Sciences AG (Lausen, CH) whereas HDAC4, 5, 7, 9, and 10 were produced as described (3). Human HDAC8 was produced as described (4). The inhibitors were tested in an enzymatic in vitro assay using 384-well plates (GreinerONe, catalogue no. 784900) (2, 4). After 5 minutes of incubation of the inhibitors with the respective enzymes (HDAC1 = 10 nM, HDAC2 and 3 = 3 nM, HDAC4 = 5 nM, HDAC5 = 10 nM, HDAC6 = 1 nM, HDAC7 = 5 nM, HDAC8 = 2 HDAC 9 = 20 nM, HDAC10 = 5 nM), the reactions were always started by the addition of substrate.

For HDAC1, 2, 3, and 6, an acetylated peptide substrate derived from p53 (Ac-RHKK(Acetyl)-AMC) was used in a discontinuous fluorescence assay(3). All reactions were performed in assay buffer (20 mM HEPES, 140 mM NaCl, 10 mM MgCl2, 1 mM TCEP and 0.2 mg/mL BSA, pH 7.4 adjusted with NaOH) at 37 °C. The reaction was quenched after 1 hour by adding trypsin and SAHA. The fluorescence intensity was measured after 1 hour of incubation using an Envision 2104 Multilabel Plate Reader (PerkinElmer, Waltham, MA), with an excitation wavelength of 380 ± 8 nm and an emission wavelength of 430 ± 8 nm. HDAC4, 5, 7, 8, 9, and 10 were measured in a continuous manner using the thio-acetylated peptide substrate (Abz-SRGGK(thio-TFA)FFRR-NH2) (3). For HDAC10, an internal quenched spermidine-like substrate was utilized. The fluorescence increase was followed for 1 hour with two reads per min with an excitation wavelength of 320 ± 8 nm and an emission wavelength of 430 ± 8 nm. Positive (enzyme, substrate, DMSO and buffer) and negative (substrate, DMSO and Buffer) controls were included in every measurement and were set as 100 and 0 %, respectively and the measured values were normalized accordingly. All tests were done in triplicates.

**Analytical data of PZ48**


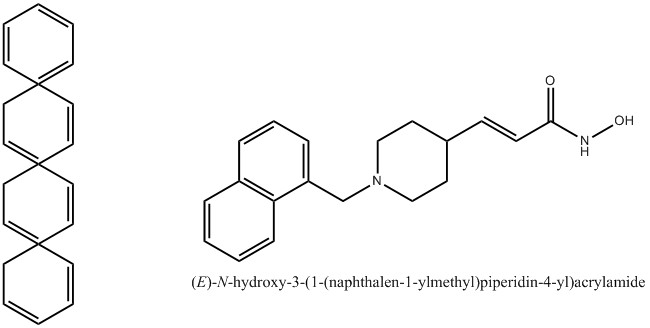


PZ48 was synthesized and purified as described in (5).


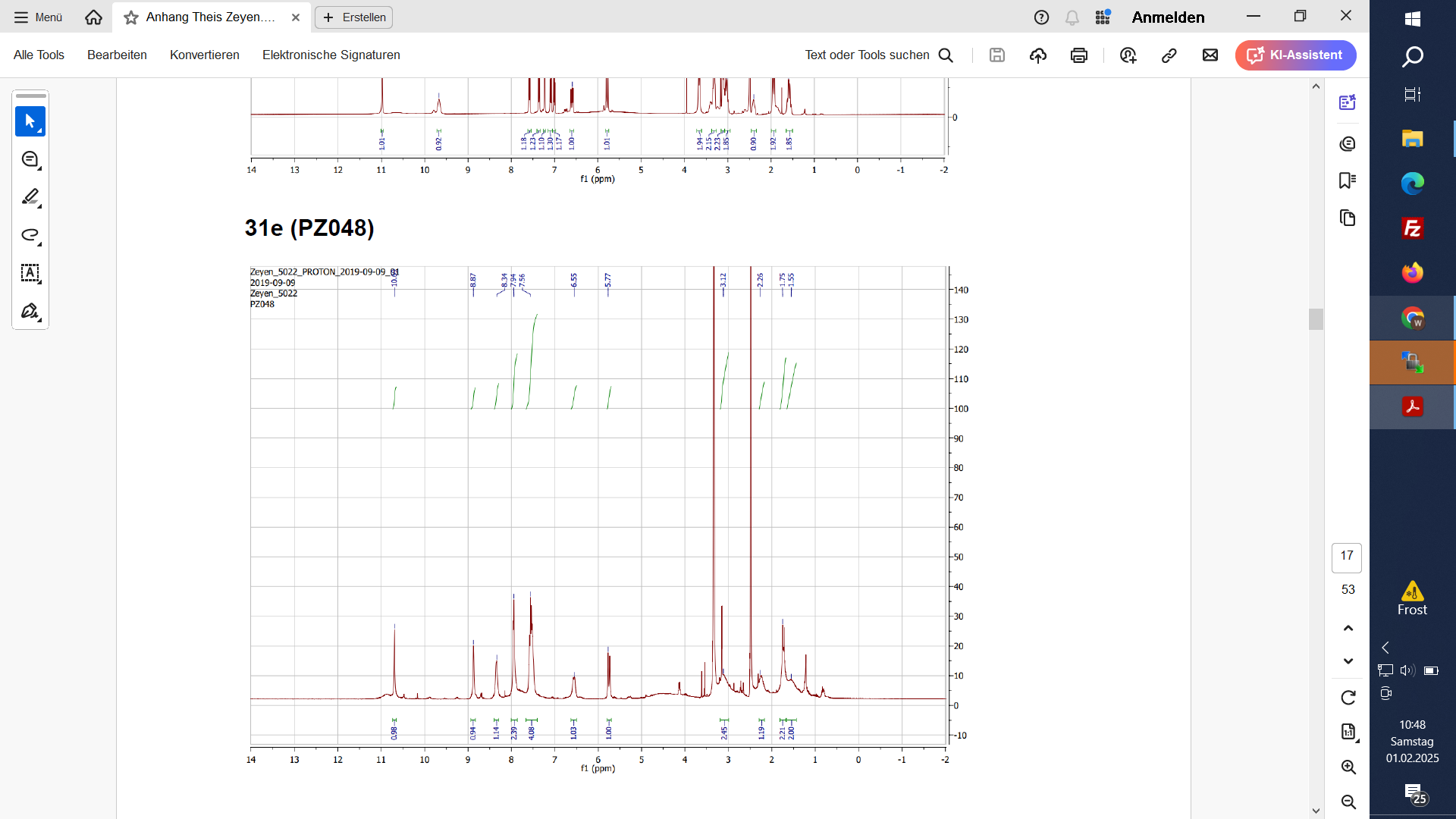


^1^H-NMR spectrum of PZ48

^1^H-NMR [(CD_3_)_2_SO, ppm]: δ = 1,55 (m, 2H, Piper_C3_-***H_2_*** & Piper_C5_-***H_2_***), 1,75 (m, 2H, Piper_C3_-***H_2_*** & Piper_C5_-***H_2_***), 2,26 (m, 1H, Piper_C4_-***H***), 3,12 (m, 2H, Piper_C2_-***H*** & Piper_C6_-***H***), 5,77 (d, 1H, CH=C***H***-CO, J_1_ = 15,6 Hz), 6,55 (m, 1H, C***H***=CH-CO), 7,56 (m, 4H, Ar_C2_-***H***, Ar_C3_-***H***, Ar_C6_-***H*** & Ar_C7_-***H***), 7,94 (m, 2H, Ar_C5_-***H*** & Ar_C8_-***H***), 8,34 (s, 1H, Ar_C4_-***H***), 8,87 (s, 1H, CO-N***H***-OH), 10,69 (s, 1H, NH-O***H***).


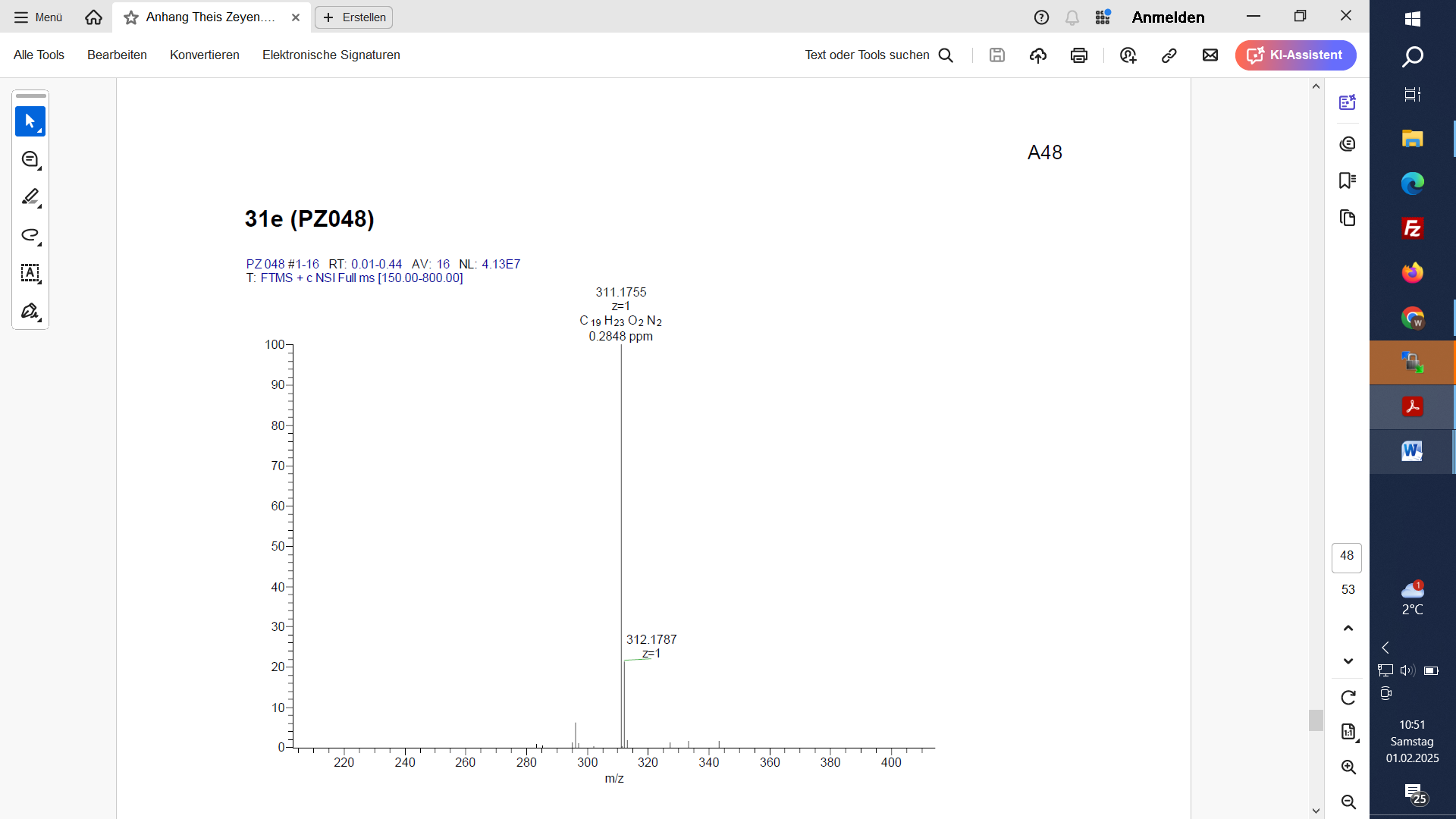


HRMS spectrum of PZ48

HR-MS m/z: 311,1755 [M+H]^+^; calculated for C_19_H_23_N_2_O_2_^+^: 311,1760


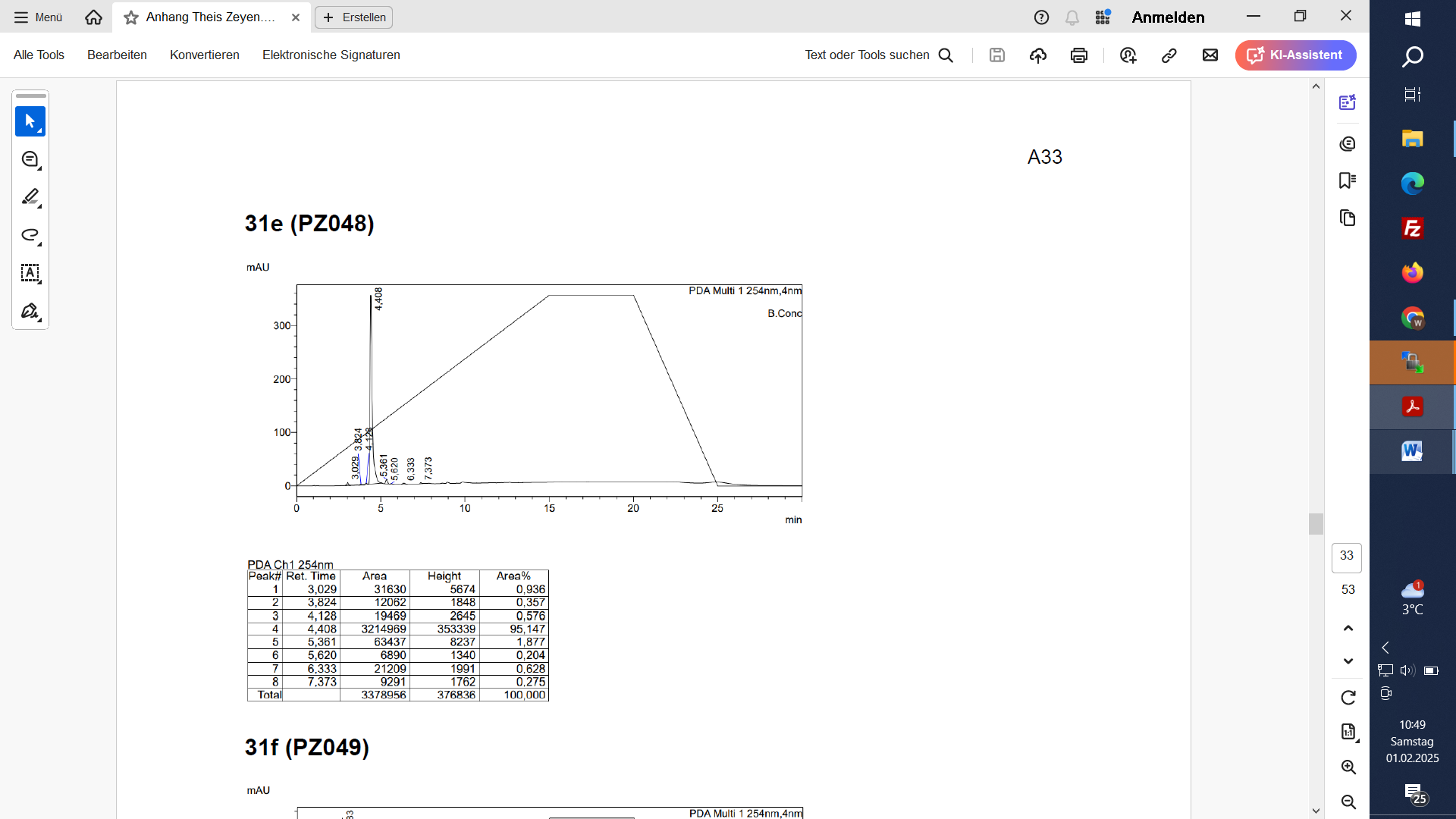


HPLC chromatogram of PZ48

HPLC: rt 4,41 min (95,15 %)

**Culture of cell lines and primary cells**

Leukemic cells were initially sourced from DSMZ and authenticated at the Leibniz-Institute DSMZ, Braunschweig, Germany, using DNA fingerprint profiling with eight highly polymorphic short tandem repeats, or from Prof. Dr. G. Winter, Vienna, Austria, as verified MOLT-4 cells. Buffy coats for the isolation of peripheral blood mononuclear cells (PBMCs) were supplied by the Blood Transfusion Unit of the University Medical Center Mainz, with analyses that are summarized in (6). Cell lines were cultured in a humidified atmosphere at 37°C with 5% CO₂, using complete medium containing RPMI-1640, 5-10% fetal bovine serum (FCS, Lonza, Cologne, Germany), and 1% penicillin/streptomycin (Sigma, Munich, Germany). Cells were confirmed to be mycoplasma-free using the MycoStrip Mycoplasma Detection Kit (InvivoGen, San Diego, California). **Table S1** lists the cell lines and their respective descriptions, taken from <https://www.dsmz.de/>, <https://www.atcc.org/>.

**Table S1:** names and descriptions of cultured cell lines

| **Cell line name** | **Description** |
| --- | --- |
| RS4-11 | B cell precursor leukemia, established from the bone marrow of a 32-year-old woman with acute lymphoblastic leukemia in first relapse |
| MOLT-4 | T cell leukemia, established from the peripheral blood of a 19-year-old man with acute lymphoblastic leukemia in relapse in 1971 |
| Ramos | Burkitt lymphoma, established from the ascitic fluid of a 3-year-old boy with American-type Burkitt lymphoma in 1972 |
| MV4-11 | established from a 10-year-old boy with acute monocytic leukemia at diagnosis |
| MOLM-13 | established from the peripheral blood of a 20-year-old man with acute myeloid leukemia AML at relapse in 1995 after initial myelodysplastic syndrome |
| HL-60 | established from the peripheral blood of a 36-year-old woman with acute myeloid leukemia in 1976 |
| RPE-1 | retinal pigment epithelial cells, established from a healthy human donor |

**Patient-derived xenograft (PDX) samples**

PDX samples were generated by (intravenously) injecting leukemia cells, isolated from patients **(Table S2)** into immune deficient NOD.Cg-Prkdcscid Il2rgtm1Wjl/SzJ or NSG mice aged 8-12 weeks as described before (7, 8). Leukemia cells were isolated from the spleen or bone marrow of the mice. If the proportion of human cells was below 90%, a mouse cell depletion kit (Miltenyi Biotec) was used to enrich the human cell population. Following enrichment, leukemia cells with ≥ 90% human cell content, derived from the bone marrows and spleens, were used for a short-term functional assay. PDX-ALL cells were cultured short-term in RPMI-1640 GlutaMax supplemented with 15% FBS, 0.1 mM 2-mercaptoethanol, 1 mM sodium pyruvate, and gentamicin (0.5 µg/ml). All animal experiments were performed in compliance with the regulatory guidelines of LANUV (Akt. 81-02.04.2017.A441) and were approved by the animal research institute (ZETT) at Heinrich Heine University Düsseldorf. Patient samples were collected with informed consent, in accordance with the Declaration of Helsinki, and the experiments were approved by the ethics committee of the medical faculty at Heinrich Heine University (Study Nr.: 2019-566).

***Table S2:*** *clinical characteristics of the B cell progenitor (BCP)-ALL patient cells xenografted in NSG mice*

| **Patient data** | | | | | **Xenograft data** | |
| --- | --- | --- | --- | --- | --- | --- |
| **Patient** | **Age at diagnosis (years)** | **Sex** | **Cytogenetics** | **MRD risk** | **% BM/ spleen blasts** | **Sacrificed after injection**  **(days)** |
| PDX-ALL-1 (initial) | 10.1 | M | *KMT2a*-r | HR | 91, 89 | 92, 116 |
| PDX-ALL1 (relapse) | N/A | M | *KMT2a*-r | N/A | 89, 87 | 88, 97 |
| PDX-ALL- 2 | 14.5 | F | *TCF3::HLF1* | HR | 97, 91 | 94, 104 |
| PDX-ALL-3 | 7.4 | F | *BCR::ABL1* | HR | 99, 91 | 110, 73 |

N/A: data not available, HR: high risk

**SILAC cell culture and mass spectrometry**

RS4-11 cells were cultured for 14 days at 37°C and 5% CO_2_ in humidified atmosphere in RPMI-1640 Medium for SILAC (ThermoFisher), which was supplied with 10% dialyzed FCS, 1% penicillin/streptomycin (Sigma, Munich, Germany) and L-Lysine/L-Arginine and sterile filtrated. For ‘light’-labelled cells, L-Arginine-0/L-Lysine-0 was used, for ‘heavy’-labelled cells Arginine-10/L-Lysine-8 was added **(Table S3)**. After the labeling stage, 400.000 cells per ml were seeded in 30 ml of the respective medium in culture dishes and incubated for 24 h at the conditions above. Next, the cells were treated with 15 µM PZ48 or left untreated for 8 h. The SILAC acetylomics experiment was performed in biological triplicate: in replicates #1 and #3, the PZ48 HDAC10 inhibitor treatment was applied to the ‘heavy’-labelled cells; in replicate #2, the labelling was swapped with the inhibitor treatment applied to the ‘light’-labelled cells. The cells were lysed using RIPA buffer (25 mM Tris-HCl pH 7.5, 150 mM NaCl, 1 mM EDTA, 0.5% [wt/vol] sodium deoxycholate, 1% [vol/vol] Triton X-100, 0.1% [wt/vol] SDS, protease inhibitor) and a Sonicator (UP200Ht, Hielscher) was used to shear DNA. Protein concentrations were measured by Bradford assay. Lysates from ‘light’ and ‘heavy’ labelled cells were mixed 1:1 (corresponding to 5 mg of protein each), and proteins were precipitated in acetone.

**Table S3**: description, concentration, product number and source of amino acids used for SILAC experiments

| **Amino acid** | **Description** | **Final concentration in culture media** | **Product number** | **Source** |
| --- | --- | --- | --- | --- |
| Lys-0 | L-Lysine monohydrochloride | 0.363 mM | L8662 | Sigma |
| Lys-8 | L-Lysine:2HCl (^13^C_6_, ^15^N_2_) | 0.363 mM | CNLM-291-H-1 | Cambridge Isotope Laboratories |
| Arg-0 | L-Arginine monohydrochloride | 0.181 mM | A6969 | Sigma |
| Arg-10 | L-Arginine:HCl (^13^C_6_, ^15^N_4_) | 0.181 mM | CNLM-539-H-1 | Cambridge Isotope Laboratories |

**Enzymatic protein digestion and acetyl-lysine peptide enrichment**

The precipitated protein pellets were resolubilized in 8 M urea containing 50 mM ammonium bicarbonate. Following reduction by 5 mM DTT, alkylation by 15 mM iodoacetamide in the dark and quenching by 5 mM DTT, the urea concentration was diluted to 2 M using 50 mM ammonium bicarbonate. Proteins were then digested by trypsin (protein:enzyme ratio of 100:1) at room temperature overnight. Following desalting in C18 Sep-Pak columns (Waters), peptides were eluted in 50% acetonitrile. Afterwards, the acetonitrile evaporated in a centrifugal evaporator. The resultant peptide solution was then adjusted with IAP buffer to reach 50 mM MOPS–NaOH (pH 7.2), 10 mM Na2HPO4, 50 mM NaCl (1X IAP buffer). Thereafter, the peptides of each sample were incubated with 40 µl slurry of the Acetyl Lysine Antibody Agarose Beads (ICP0388-5MG, ImmuneChem Pharmaceuticals, Burnaby, Canada) at 4°C overnight. Following sequential washes in 1X IAP buffer and water, peptides were eluted from the beads 4 times in 0.15% TFA.

**Tip-based high-pH reversed phase peptide fractionation**

The eluted peptides were desalted on C18 StageTip (Empore, 3M) (9) and fractionated on the tip in 10 mM ammonium bicarbonate at pH 8 sequentially at the following concentrations of acetonitrile: 3%, 5%, 7%, 9%, 12%, 15%, 18%, 21%, 25%, 30%, 60%. Except for the faction at 15% acetonitrile, the early and late fractions were further concatenated as the following combinations: 3% + 18%, 5% + 21%, 7% + 25%, 9% + 30%, 12% + 60%. In total, 6 peptide fractions per sample were prepared and dried to completeness in a centrifugal evaporator at 45°C. The dried peptides were reconstituted in 80% acetonitrile, 0.1% formic acid. After the acetonitrile was evaporated in a centrifugal evaporator, the peptides were acidified by adding 0.1% formic acid.

**Liquid chromatography tandem mass spectrometry**

Peptides were analyzed using an Orbitrap Exploris 480 mass spectrometer (Thermo Fisher Scientific) coupled to an EASY-nLC 1200 UHPLC system (Thermo Fisher Scientific). Peptides were separated in an in-house packed 60-cm analytical column (inner diameter: 75 μm; ReproSil-Pur 120 C18-AQ 1.9-μm silica particles, Dr. Maisch GmbH) by online reversed phase chromatography through a 90 min gradient of 2.4-32% acetonitrile with 0.1% formic acid at a nanoflow rate of 250 nl/min. The eluted peptides were sprayed directly by electrospray ionization into the mass spectrometer. Mass spectrometry measurement was conducted in data-dependent acquisition mode using a top15 method with one full scan (resolution: 60,000, scan range: 300-1650 m/z, target value: 3 × 106, maximum injection time: 40 ms) followed by 15 fragmentation scans via higher energy collision dissociation (HCD; normalized collision energy 30%; resolution: 15,000, target value: 1 × 105, maximum injection time: 40 ms, isolation window: 1.4 m/z). Only precursor ions of +2 to +8 charge state were selected for fragmentation scans. Additionally, precursor ions already isolated for fragmentation were dynamically excluded for 25 s.

**Mass spectrometry data processing and statistical analysis**

Mass spectrometry raw data files were processed using MaxQuant software (version 2.1.3.0) (10). MS/MS mass spectra were searched using Andromeda search engine (11) against a target-decoy database containing the forward and reverse protein sequences of UniProt H. sapiens reference proteome (release 2022_04; 102,601 entries) and a default list of common contaminants. Corresponding SILAC states were assigned (light: Arg0, Lys0; heavy: Arg10, Lys8). A maximum of 3 labelled amino acids per peptide was considered. Trypsin/P specificity was assigned. Carbamidomethylation of cysteine was set as fixed modification. Acetylation at lysine residue, methionine oxidation and protein N-terminal acetylation were chosen as variable modifications. A maximum of 2 missed cleavages were tolerated. The minimum peptide length was set to be 7 amino acids. The “second peptides” search was switched on. False discovery rate (FDR) was set to 1% at both peptide and protein levels.

For protein quantification, the minimum ratio count was set to one. Both the unique and razor peptides were used for quantification. The “re-quantify” function was switched on. The “advanced ratio estimation” option was also chosen. The normalized ratios (HDAC10 inhibitor/control) of the detected Acetyl (K) sites were log2-transformed (ratios of a label-swap replicate were first inverted). The log2 ratios were filtered for detection in at least 2 out of 3 replicates. A linear model was then fitted using the limma package in R (12) to assess the ratios for each site without further adjustment for multiple testing. The log2 fold change and the significance of the difference were displayed on a volcano plot. Only sites with a minimum log2 fold change of 1 and a p value lower than 0.01 were considered as being differentially regulated.

**Flow cytometry analyses**

These analyses were performed with a FACS Canto (BD Biosciences, Heidelberg, Germany). For each treatment condition, 150,000 cells/ml were seeded in 2 ml complete medium. After 24 h, cells were treated as necessary. Cells were washed with 1 ml PBS and processed as mentioned below.

**Flow cytometry-based apoptosis assay**

After a washing step with PBS, cells were resuspended in 50 µl Annexin binding buffer containing 2 µl Annexin V-FITC. After 30 min, 450 µl Annexin binding buffer containing 10 µl PI staining solution (50 µg/ml) was added, and cells were analyzed immediately. Due to morphological changes during apoptosis, Annexin V binds to the phosphatidylserines that is exposed on apoptotic cell membranes. Thus, cells in the early stages of the process are Annexin V-FITC-positive. In the later stages of the process, PI can bind to the DNA of the cell. Based on the measured fluorescence signals, cell populations were gated and measured.

The apoptosis and necrosis induction were detected in PZ48 treated PDX-ALL cells using the Annexin V-FITC apoptosis kit (Biolegend, Cat. 640906). Initially, cells were seeded with a cell density of 1x10^6^/ml in 12-well cell culture plates and treated with solvent DMSO or PZ48 (2, 5, 10 and 15 µM). After 72 h, the cells were harvested, washed once with Annexin V Binding Buffer (Biolegend, Cat. 422201) and labeled with PI and Annexin V-FITC according to the manufacturer’s protocol. After 15 min incubation time, Annexin V-FITC/PI-stained cells were analyzed via flow cytometry (Beckman Coulter CytoFLEX Cytometer) in order to identify the apoptotic and necrotic cell proportions.

**Flow cytometry-based CYTO-ID assay**

To measure a change in autophagic flux, the CYTO-ID Kit (Enzo Lifesciences) was used to stain autophagosomes. After washing, cells were resuspended in 250 µl of RPMI-1640 without phenol red and then 250 µl of RPMI-1640 without phenol red containing 1:1000 CYTO-ID stain green were added. Cells were incubated for 30 min at 37°C in the dark. After the incubation, cells were washed with 2 ml PBS, resuspended in 500 µl PBS and measured immediately. To determine an increase or decrease in autophagy, the mean FITC fluorescence was measured and compared to untreated control cells. To enhance the shift of the fluorescence, chloroquine was added to block fusion of autophagosomes and lysosomes resulting in an accumulation of autophagosomes.

**Flow cytometry-based cell cycle distribution assay**

The DNA content of a cell indicates their cell cycle phases. To measure the cell cycle distribution, cells were first fixed in 2 ml of 80% ethanol overnight. After centrifugation, 350 µl RNase A solution was added and incubated for 1 h to degrade RNA in the sample. Lastly, 150 µl PI staining solution was added, and cells were measured for the intensity of the PI signal. Doublets were excluded from the analyses by gating. Cells in G1 phase have 2n chromosomes, typically representing most of the cell population. In S phase, DNA content is rising to the peak in G2 phase, where the number of chromosomes is doubled to 4n. Fragmented DNA is measured as subG1 phase. The cell cycle distribution was analyzed using gating for the different DNA contents of the cells.

**Flow cytometry-based γH2AX assay**

To determine in which cell cycle phase DNA damage occurs, cells were harvested by centrifugation (5 min, 1300 rpm), resuspended in 200 µl PBS, and prefixed in 2 ml ice cold 1% formaldehyde for 15 min. Then, cells were washed twice with PBS. Cells were resuspended in 200 µl PBS again and 2 ml ice cold 80% ethanol was added and incubated for 10 min at -20°C. After that, cells were washed again in PBS and then rehydrated in 5 ml PBS for 5 min at room temperature. Next, cells were resuspended in 1 ml 0,25% Triton-X in PBS and incubated for 5 min on ice. After centrifugation (5 min, 1300 rpm), the pellet was resuspended in 100 µl 1% BSA in PBS containing anti-γH2AX primary antibody **(Table S4)** and incubated over night at 4°C with gentle agitation. The next day, 1 ml of 1% BSA was added, and cells were centrifuged. After centrifugation, the pellet was resuspended in 100 µl 1% BSA in PBS containing 1:33 of secondary antibody Alexa Fluor 488 (Life Technologies) and incubated for 30 min in the dark at room temperature. After washing with 1% BSA in PBS, cells were resuspended in 500 µl PBS containing 10 µg/ml PI and 100 µg/ml RNase. After 30 min of incubation at 37°C in the dark, fluorescence was measured immediately.

**Immunoblot and antibodies**

After washing with PBS, cells were lysed using NET-N buffer (100 mM NaCl, 10 mM Tris-HCl pH 8, 1 mM EDTA, 10% glycerin, 0.5% NP-40 plus cOmplete protease inhibitor tablets (Roche) and phosphatase inhibitor cocktail 2 (Sigma)) for 30 min on ice and sonicated (10 s/40% amplitude). Protein concentrations were measured by Bradford assay. Proteins were transferred to nitrocellulose membranes (0.2 μm, Amersham Protran, GE Healthcare) and processed for immunoblotting. Detection of proteins was performed with the Odyssey Infrared Imaging System (Licor). Antibodies used for immunoblotting are shown in **Table S4**.

To ensure the detection of H10, we used a home-mad antibody.

For HDAC10 protein expression analyses, a monoclonal anti-HDAC10 antibody was generated with the support of the Core Facility Antibodies of the German Cancer Research Center (DKFZ), according to the principles of Köhler and Milstein's hybridoma technology (13). In brief, C57BL/6N mouse immunization was performed with purified 6His-HDAC10 protein fragment (aa 500-617). Anti‑HDAC10 antibody-producing B-lymphocytes isolated from positively reacted mice were fused with Sp2/0 murine myeloma cells (RRID: CVCL_2199) to produce hybridoma cell clones. The supernatant from validated monoclonal clones was used for immunodetection of HDAC10 protein.

**Table S4**: antibodies and further information

| **Antibody** | **Source** | **Product No.** | **Dilution** |
| --- | --- | --- | --- |
| ac-HDAC3 | Merck KGaA | 06-599 | 1:2000 |
| ac-SMC3 | Merck KGaA | MABE1925 | 1:1000 |
| ac-Tubulin | Merck KGaA | T7451 | 1:2000 |
| cl. Caspase-3 | Cell Signaling Tech. | 96615 | 1:1000 |
| GAPDH | Santa Cruz Biotech. | sc-32233 | 1:5000 |
| HDAC10 | DKFZ Heidelberg | HDAC10T2 #50/7/1 | - |
| HDAC8 | Santa Cruz Biotech. | sc-374180 | 1:1000 |
| HSP90 | Santa Cruz Biotech. | sc-13119 | 1:1000 |
| MYC | Cell Signaling Tech. | 5605 | 1:500 |
| PAR | Merck KGaA | MABE1031 | 1:1000 |
| PARG | Santa Cruz Biotech. | sc-398563 | 1:1000 |
| PARP1 | BD Pharmingen™ | 556362 | 1:2000 |
| pCHK1 (S345) | Cell Signaling Tech. | 2348 | 1:1000 |
| pCHK2 (T68) | Cell Signaling Tech. | 2661 | 1:1000 |
| POLD1 | Santa Cruz Biotech. | sc-374025 | 1:100 |
| p-RPA32 (S33) | Bethyl Laboratories | A300-246A | 1:800 |
| Ubiquitin | Merck KGaA | 05-1307 | 1:1000 |
| β-Actin | Santa Cruz Biotech. | sc-47778 | 1:2000 |
| β-Tubulin | Merck KGaA | T4026 | 1:1000 |
| γH2AX | Cell Signaling Tech. | 9718 | 1:1000 |

**Immunofluorescence microscopy**

For immunofluorescence microscopy, 150.000 cells/ml were seeded in 2 ml of RPMI-1640 medium 24 h prior to the treatment. After treatment, the cells were centrifuged for 5 min at 1300 rpm and the supernatant was removed. The cells were then washed with 1 ml of PBS and centrifuged again for 5 min at 1300 rpm. For permeabilization, the pellet was resuspended in 1 ml of a 2% formaldehyde solution and incubated for 15 min at room temperature. After washing again with PBS, cells were resuspended in 20 µl of PBS and dried on cover slips which were cleaned with ethanol and 1 M HCl before. For fixation of the cells, 2 ml of methanol/acetone (7:3) were added and the cells were incubated for 9 min at -20°C. After that, the cells were washed thrice with PBS for 5 min. To block unspecific binding sites, 200 µl of PBS with 5% BSA and 0,25% Triton X-100 were added to the cover slips and incubated for 1 h at room temperature. After that, 150 µl of the blocking solution above combined with the respective primary antibody **(Table S4)** were added and incubated overnight at 4°C in a wet chamber. On the next day, the cover slips were washed thrice with PBS for 5 min and 150 µl of the blocking solution above combined with Alexa Fluor 488 goat anti-rabbit or anti-mouse (1:300, Life technologies) were added. The coverslips were incubated in the dark for 1 h. After incubation, the cover slips were washed twice with PBS for 5 min, then 2 min with PBS high salt (PBS+0.4 M NaCl) and 5 min with PBS again. To stain the nuclei, 100 µl of Vectashield® (Vector Laboratories) with TO-PRO-3 (1:100, Life technologies) were added and the cover slips were sealed with nail polish onto slides and measured immediately. To analyze the samples and capture images, a Zeiss Axio Observer.Z1 microscope, with a LSM710 laser-scanning unit (Zeiss) was used.

**PBMCs**

PBMCs were isolated from buffy coats (Blood transfusion unit of the University Medical Center Mainz) and analyzed as described by us recently (14). Antibodies for lineage-specific cell surface markers: CD3−CD19+, B cells; CD3+, T cells, CD3−CD19−CD14+, monocytes; CD3−CD19−CD1c+, dendritic cells; CD3−CD19−CD56+, natural killer (NK) cells; CD3−CD14−CD19−CD56−CD11b+, PMNs. Cell viability was evaluated using Annexin-V AF647 (#A23204; early apoptosis marker) and FVD eFl780 (#65-0865-18; late apoptosis marker; both from ThermoFisher). The following antibodies were used: CD11b BV510 (#101263), CD1c BV605 (#331538), CD3 BV711 (#344838) from BioLegend, San Diego, CA, USA; CD14 PE-eFl610 (#61-0149-42), CD56 Pe-Cy7 (#25-0567-42), CD19 AF488 (#53-0199-42) from ThermoFisher.

**T and B cell activation**

PBMCs were incubated with R848 (1 ug/ml) or Dynabeads™ Human T-Activator CD3/CD28 (5 µl / 1 ml cell suspension) for 24 h. Then, PBMCs were treated with increasing doses (5 µM, 10 µM, 15 µM) of PZ48 for 24 h. Staining of FVD eFl780 was analyzed using flow cytometry (*n*=5, mean+SD; two-way ANOVA; n.s., not significant). T cells were defined as CD3+, B cells were defined as CD3-CD19+.

**Single cell gel electrophoresis**

Single cell electrophoresis was used to determine single strand breaks in the DNA of isolated cells. Ten µl of a 10^6^ cells/ml suspension were mixed with 120 µl low melting point agarose on a slide with a coverslip and left for 5 min at 4°C. Then, the coverslips were removed, and the slides were put into lysis buffer (2,5 M NaCl, 100 mM EDTA, 10 mM Tris, 1% Na-laurylsarcosinat, 1% Triton X-100, 10% DMSO, pH10) for 1 h. After that, the slides were incubated in electrophoresis buffer (300 mM NaOH, 1 mM EDTA, pH13) for alkaline denaturation at 4°C for 25 min. Electrophoresis was done for 25 min with 25 V and 300 mA at 4°C. Lastly, slides were incubated in neutralization buffer (0,4 M Tris, pH 7,5) for 5 min following an incubation in EtOH for 5 min. The DNA was stained with ethidium bromide for visual analysis.

**Colony formation assay**

Colony formation assay of human HSPCs derived from healthy donors was performed as previously described (15, 16). In brief, 1x10^5^ cells per sample were resuspended in 500 µl SFEM medium (Stemcell Technologies) and mixed with 3.5 ml methylcellulose (Stemcell Technologies, H4034^Optimum^). Depending on the condition, 5 µM PZ48 or the respective amount of DMSO was added to the mixture and vortexed. The mixture was transferred into 3 cm culture dish plates. The plates were incubated at 37°C and 5% CO_2._ After 10 days, colonies were counted, and pictures were taken.

For RS4-11 cells, 1500 cells per sample were washed in PBS and centrifuged (5 min, 1300 rpm). The cells were then resuspended in 100 µl resuspension buffer (R&D Systems, Minneapolis, Minnesota, USA) and mixed with 900 µl methylcellulose (R&D Systems, Minneapolis, Minnesota, USA). Depending on the condition, 5 µM PZ48 or the respective amount of DMSO was added to the mixture and vortexed. After 20 min, the mixture was transferred into 3 cm culture dish plates. The plates were incubated at 37°C and 5% CO_2._ After 10 days, colonies were counted, and pictures were taken.

**RNA sequencing**

NGS library prep was performed with Illumina's Stranded mRNA Prep Ligation Kit following the Stranded mRNA Prep Ligation ReferenceGuide (June 2020) (Document # 1000000124518 v00). Libraries were prepared with a starting amount of 1000 ng and amplified in 9 PCR cycles. After post-PCR purification, an additional round of purification was performed using 1x of AMPure XP beads. Libraries were profiled in a High Sensitivity DNA on a 2100 Bioanalyzer (Agilent technologies) and quantified using the Qubit dsDNA HS Assay Kit, in a Qubit 2.0 Fluorometer (Life technologies). The samples (two replicates of untreated lymphoblast cell line (RS411) and two replicates of PZ48 treated cells) were pooled in an equimolar ratio and sequenced on 1 NextSeq500 Highoutput FC, SR for 1x79 cycles plus 10 cycles for the index read and 2 dark cycles upfront R1.

Reads were aligned on the *Homo sapiens* genome assembly hg38 (ENSEMBL release 98) using STAR (version 2.7.10a, parameters: --outMultimapperOrder Random --outSAMattributes NH HI AS nM MD –outSJfilterReads Unique --outSAMunmapped Within --outFilterMismatchNoverReadLmax 0.04 –outFilterMismatchNmax 999 –sjdbOverhang 79) (17). The featureCounts program (version 2.0.0, parameter**:** -s2) was used to count the number of reads overlapping genes (18). Differentially expressed genes (DEGs) were determined using DESeq2 (release: 1.34.0) with corrected pvalue (Benjamini and Hochberg, FDR) < 0.05 (19). The KEGG pathway enrichment analysis and Gene Set Enrichment Analysis (GSEA) were performed using the enrichKEGG and gseKEGG functions of the clusterProfiler package (release: 4.10.0) (20).

**Zebrafish experiments**

**Cell preparation for *Danio rerio* embryo xenotransplantation**

To determine the number of viable RS4-11 cells, 1 mL of cell suspension was analyzed using the automated ViCell cell counter, which employs trypan blue staining to distinguish viable from non-viable cells. Next, 1 × 10⁶ viable cells/mL were stained with 5 µL of CM-DiD (CellTracker CM-DiD; Thermo Fisher Scientific, Waltham, MA, USA) according to the manufacturer’s protocol. Briefly, cells were incubated at 37 °C for 5 minutes, followed by centrifugation and two washes with FCS-free RPMI-1640 medium (without phenol red). After a final centrifugation step, cells were resuspended at a concentration of 1.5 × 10⁶ cells/10 µL. The cell suspension was filtered through a 40 µm cell strainer to remove cell clumps.

During injections, cells were maintained on ice. Embryos were anesthetized with tricaine (MS-222, tricaine methanesulfonate, 0.02% w/v; Sigma-Aldrich, Munich, Germany; 1× tricaine) prior to injection. Microinjection needles (BioMedical Instruments) were prepared by cutting the pipette tip with a scalpel under a microscope to achieve an opening suitable for the cell size.

Approximately 8–10 µL of the cell suspension was loaded into each capillary and injected into the yolk sac of the embryos using the Eppendorf FemtoJet express microinjector (Eppendorf, Hamburg, Germany) and an Eppendorf Micromanipulator. After injection, embryos were maintained at 34 °C, and tumor cell fluorescence in the yolk sac was assessed via fluorescence imaging at 1–2 hours post-injection (hpi). On average, 70–80% of embryos were successfully injected with fluorescently labeled cells.

Before treatment initiation, the zebrafish patient-derived xenografts were sorted into groups based on mean tumor sizes, ensuring that each group contained a mix of small, medium, and large tumors.

**Drug treatment of *Danio rerio* embryos**

Drugs were administered in 1× E3 buffer supplemented with 0.2 mM PTU. Treatment began after the initial imaging time point at 24 hours post-injection (hpi) (baseline) and continued for 48 hours, concluding at 72 hpi. It is generally estimated that zebrafish embryos absorb approximately 1/20 to 1/10 of the applied drug concentrations (21, 22).

**Imaging and analysis of zebrafish embryos bearing human tumor cells**

Imaging was performed at 24 hours post-injection (hpi) and 72 hpi at a temperature of 32–34 °C using the ImageXpress Micro Confocal High-Content Microscope (Molecular Devices, San José, CA, USA). Embryos were anesthetized with 1× tricaine and placed individually into Hashimoto zebrafish 96-well plates (Funakoshi Co., Ltd., Tokyo, Japan), with one embryo per well. Images were captured at 4× magnification in multiple z-stacks (35 z-stacks per image; step size: 20 µm) from two sites per well, covering the entire yolk sac, using brightfield and Cy5 settings.

Tumor volume was quantified using an automated analysis pipeline (an in-house ImageJ macro), ensuring accurate discrimination of specific tumor signals from background noise caused by the yolk sac, as previously described (23). To assess tumor growth or response to treatment, the percentage change in tumor volume was calculated between the baseline (24 hpi) and the end of treatment (72 hpi). The zebrafish-adapted Response Evaluation Criteria in Solid Tumors (RECIST), as described previously (23), was used to evaluate drug response:

Progressive disease (PD): Tumor volume increase of at least 20%.

Partial response (PR): Tumor volume decrease of more than 30%.

**Databases**

The human protein atlas (<https://www.proteinatlas.org>, (24)) was used to asses *HDAC10* mRNA levels in cell lines. We searched for HDAC10 and the category Cell line.

The interactive online resource Hemap (<http://hemap.uta.fi/hemap/index.html>; (25)) contains curated genome-wide data across different diseases of the hematopoietic system. We used the grid GEXP Boxplots to plot *HDAC10* using the cancer categories ALL and AML.

Enrichr (<https://maayanlab.cloud/Enrichr/>; (26-28)) was used for GSEA using the differently regulated genes from RNA sequencing.

**Schematic illustrations**

The graphical abstract was created in BioRender. Mohamed, A. (2025) <https://BioRender.com/vff97af>. Figure 5C was created in BioRender. Mieland, A. (2025) <https://BioRender.com/t85m878>.

**Quantification and statistical analysis**

Data analysis was conducted using GraphPad Prism 8. Statistical significance was assessed via t-test or one-way/two-way ANOVA. Multiple comparisons were made with Bonferroni correction. Asterisks indicate p-values (∗p ≤ 0.05; ∗∗p ≤ 0.01; ∗∗∗p ≤ 0.001; ∗∗∗∗p ≤ 0.0001). If not stated otherwise, the experiments were done as biological triplicates. Specifics of the statistical methods are provided in the figure legends.

**References**

1. Kutil Z, Novakova Z, Meleshin M, Mikesova J, Schutkowski M, Barinka C. Histone Deacetylase 11 Is a Fatty-Acid Deacylase. ACS Chem Biol. 2018;13:685-93.

2. Baselious F, Hilscher S, Robaa D, Barinka C, Schutkowski M, Sippl W. Comparative Structure-Based Virtual Screening Utilizing Optimized AlphaFold Model Identifies Selective HDAC11 Inhibitor. Int J Mol Sci. 2024;25.

3. Zessin M, Kutil Z, Meleshin M, Novakova Z, Ghazy E, Kalbas D, et al. One-Atom Substitution Enables Direct and Continuous Monitoring of Histone Deacylase Activity. Biochemistry. 2019;58:4777-89.

4. Heimburg T, Kolbinger FR, Zeyen P, Ghazy E, Herp D, Schmidtkunz K, et al. Structure-Based Design and Biological Characterization of Selective Histone Deacetylase 8 (HDAC8) Inhibitors with Anti-Neuroblastoma Activity. J Med Chem. 2017;60:10188-204.

5. Zeyen P, Zeyn Y, Herp D, Mahmoudi F, Yesiloglu TZ, Erdmann F, et al. Identification of histone deacetylase 10 (HDAC10) inhibitors that modulate autophagy in transformed cells. Eur J Med Chem. 2022;234:114272.

6. Zeyn Y, Hausmann K, Halilovic M, Beyer M, Ibrahim HS, Brenner W, et al. Histone deacetylase inhibitors modulate hormesis in leukemic cells with mutant FMS-like tyrosine kinase-3. Leukemia. 2023;37:2319-23.

7. Oikonomou A, Valsecchi L, Quadri M, Watrin T, Scharov K, Procopio S, et al. High-throughput screening as a drug repurposing strategy for poor outcome subgroups of pediatric B-cell precursor Acute Lymphoblastic Leukemia. Biochem Pharmacol. 2023;217:115809.

8. Vogt M, Dienstbier N, Schliehe-Diecks J, Scharov K, Tu JW, Gebing P, et al. Co-targeting HSP90 alpha and CDK7 overcomes resistance against HSP90 inhibitors in BCR-ABL1+ leukemia cells. Cell Death Dis. 2023;14:799.

9. Rappsilber J, Ishihama Y, Mann M. Stop and go extraction tips for matrix-assisted laser desorption/ionization, nanoelectrospray, and LC/MS sample pretreatment in proteomics. Anal Chem. 2003;75:663-70.

10. Cox J, Mann M. MaxQuant enables high peptide identification rates, individualized p.p.b.-range mass accuracies and proteome-wide protein quantification. Nat Biotechnol. 2008;26:1367-72.

11. Cox J, Neuhauser N, Michalski A, Scheltema RA, Olsen JV, Mann M. Andromeda: a peptide search engine integrated into the MaxQuant environment. J Proteome Res. 2011;10:1794-805.

12. Phipson B, Lee S, Majewski IJ, Alexander WS, Smyth GK. Robust Hyperparameter Estimation Protects against Hypervariable Genes and Improves Power to Detect Differential Expression. Ann Appl Stat. 2016;10:946-63.

13. Köhler G, Milstein C. Continuous cultures of fused cells secreting antibody of predefined specificity. Nature. 1975;256:495-7.

14. Kansy AG, Ashry R, Mustafa AM, Alfayomy AM, Radsak MP, Zeyn Y, et al. Pharmacological degradation of ATR induces antiproliferative DNA replication stress in leukemic cells. Mol Oncol. 2024;18:1958-65.

15. Perner F, Schnoeder TM, Xiong Y, Jayavelu AK, Mashamba N, Santamaria NT, et al. YBX1 mediates translation of oncogenic transcripts to control cell competition in AML. Leukemia. 2022;36:426-37.

16. Schnoeder TM, Schwarzer A, Jayavelu AK, Hsu CJ, Kirkpatrick J, Dohner K, et al. PLCG1 is required for AML1-ETO leukemia stem cell self-renewal. Blood. 2022;139:1080-97.

17. Dobin A, Davis CA, Schlesinger F, Drenkow J, Zaleski C, Jha S, et al. STAR: ultrafast universal RNA-seq aligner. Bioinformatics. 2013;29:15-21.

18. Liao Y, Smyth GK, Shi W. The Subread aligner: fast, accurate and scalable read mapping by seed-and-vote. Nucleic Acids Res. 2013;41:e108.

19. Love MI, Huber W, Anders S. Moderated estimation of fold change and dispersion for RNA-seq data with DESeq2. Genome Biol. 2014;15:550.

20. Yu G, Wang LG, Han Y, He QY. clusterProfiler: an R package for comparing biological themes among gene clusters. OMICS. 2012;16:284-7.

21. Wertman J, Veinotte CJ, Dellaire G, Berman JN. The Zebrafish Xenograft Platform: Evolution of a Novel Cancer Model and Preclinical Screening Tool. Adv Exp Med Biol. 2016;916:289-314.

22. Fior R, Povoa V, Mendes RV, Carvalho T, Gomes A, Figueiredo N, et al. Single-cell functional and chemosensitive profiling of combinatorial colorectal therapy in zebrafish xenografts. Proc Natl Acad Sci U S A. 2017;114:E8234-E43.

23. Wrobel JK, Najafi S, Ayhan S, Gatzweiler C, Krunic D, Ridinger J, et al. Rapid In Vivo Validation of HDAC Inhibitor-Based Treatments in Neuroblastoma Zebrafish Xenografts. Pharmaceuticals (Basel). 2020;13.

24. Uhlen M, Karlsson MJ, Zhong W, Tebani A, Pou C, Mikes J, et al. A genome-wide transcriptomic analysis of protein-coding genes in human blood cells. Science. 2019;366.

25. Polonen P, Mehtonen J, Lin J, Liuksiala T, Hayrynen S, Teppo S, et al. Hemap: An Interactive Online Resource for Characterizing Molecular Phenotypes across Hematologic Malignancies. Cancer Res. 2019;79:2466-79.

26. Chen EY, Tan CM, Kou Y, Duan Q, Wang Z, Meirelles GV, et al. Enrichr: interactive and collaborative HTML5 gene list enrichment analysis tool. BMC Bioinformatics. 2013;14:128.

27. Kuleshov MV, Jones MR, Rouillard AD, Fernandez NF, Duan Q, Wang Z, et al. Enrichr: a comprehensive gene set enrichment analysis web server 2016 update. Nucleic Acids Res. 2016;44:W90-7.

28. Xie Z, Bailey A, Kuleshov MV, Clarke DJB, Evangelista JE, Jenkins SL, et al. Gene Set Knowledge Discovery with Enrichr. Curr Protoc. 2021;1:e90.
