# Supplementary material for: The protein deacetylase HDAC10 controls DNA replication in malignant lymphoid cells
Source: Leukemia. 2025 Apr 29;39(7):1756–68. doi: 10.1038/s41375-025-02612-8 (PMC12208866; doi:10.1038/s41375-025-02612-8)
Supplement: Supplementary file 2 — Figs. S1-S5 [file 41375_2025_2612_MOESM2_ESM.ppt]

## Slide 1
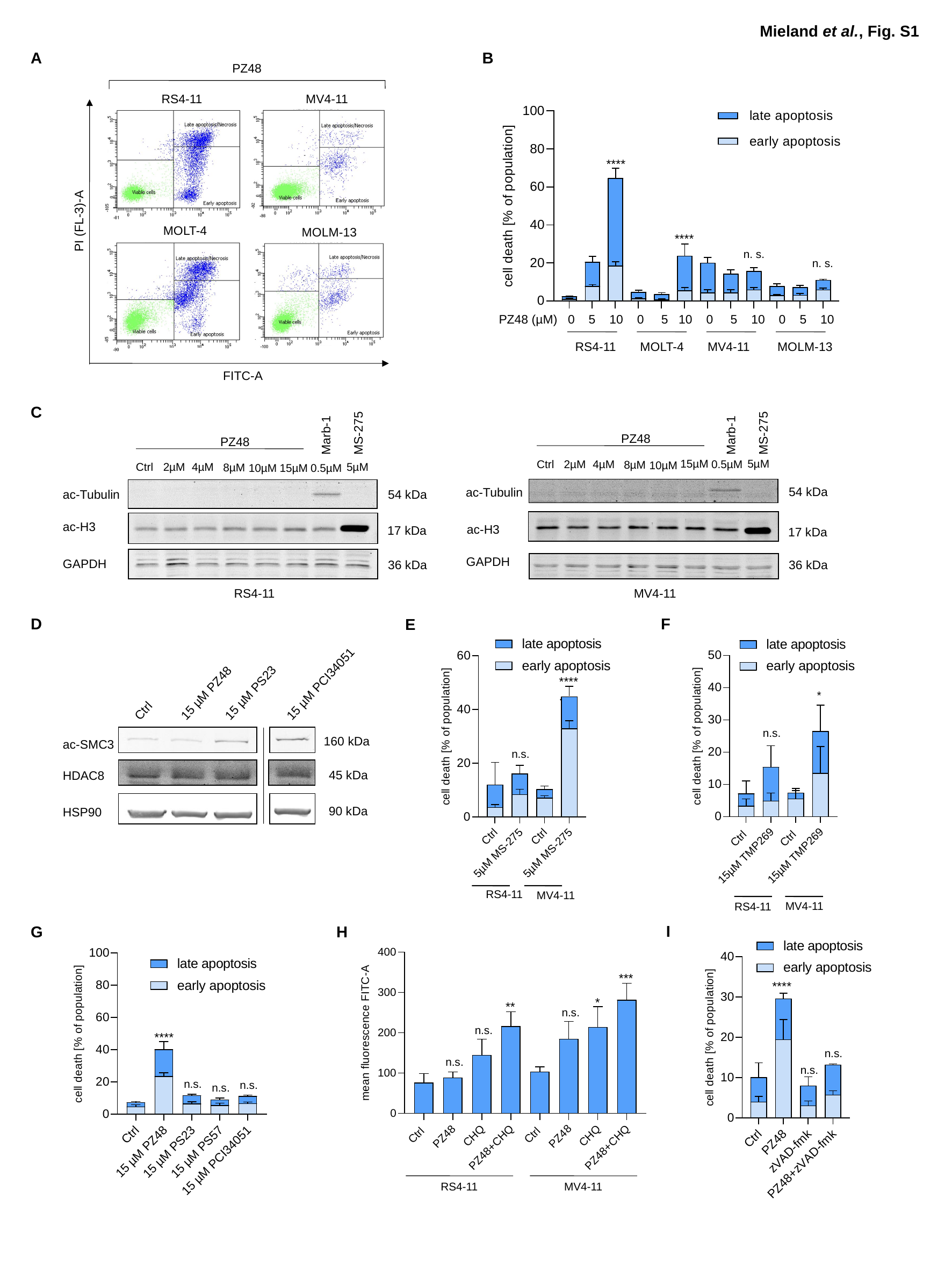

Mieland et al., Fig. S1
B
A
PZ48
RS4-11
MV4-11
****
PI (FL-3)-A
MOLT-4
MOLM-13
****
n. s.
n. s.
PZ48 (µM) 0 5 10 0 5 10 0 5 10 0 5 10
RS4-11
MOLT-4
MV4-11
MOLM-13
FITC-A
C
MS-275
MS-275
Marb-1
Marb-1
PZ48
PZ48
15µM
5µM
Ctrl
2µM
4µM
0.5µM
8µM
10µM
Ctrl
2µM
4µM
5µM
8µM
0.5µM
15µM
10µM
54 kDa
ac-Tubulin
ac-Tubulin
54 kDa
ac-H3
ac-H3
17 kDa
17 kDa
GAPDH
GAPDH
36 kDa
36 kDa
RS4-11
MV4-11
D
F
E
15 µM PCI34051
****.
15 µM PS23
15 µM PZ48
*
Ctrl
n.s.
160 kDa
ac-SMC3
n.s.
45 kDa
HDAC8
90 kDa
HSP90
RS4-11
MV4-11
MV4-11
RS4-11
I
G
H
***
****
*
**
n.s.
n.s.
****
n.s.
n.s.
n.s.
n.s.
n.s.
n.s.
RS4-11
MV4-11

## Slide 2
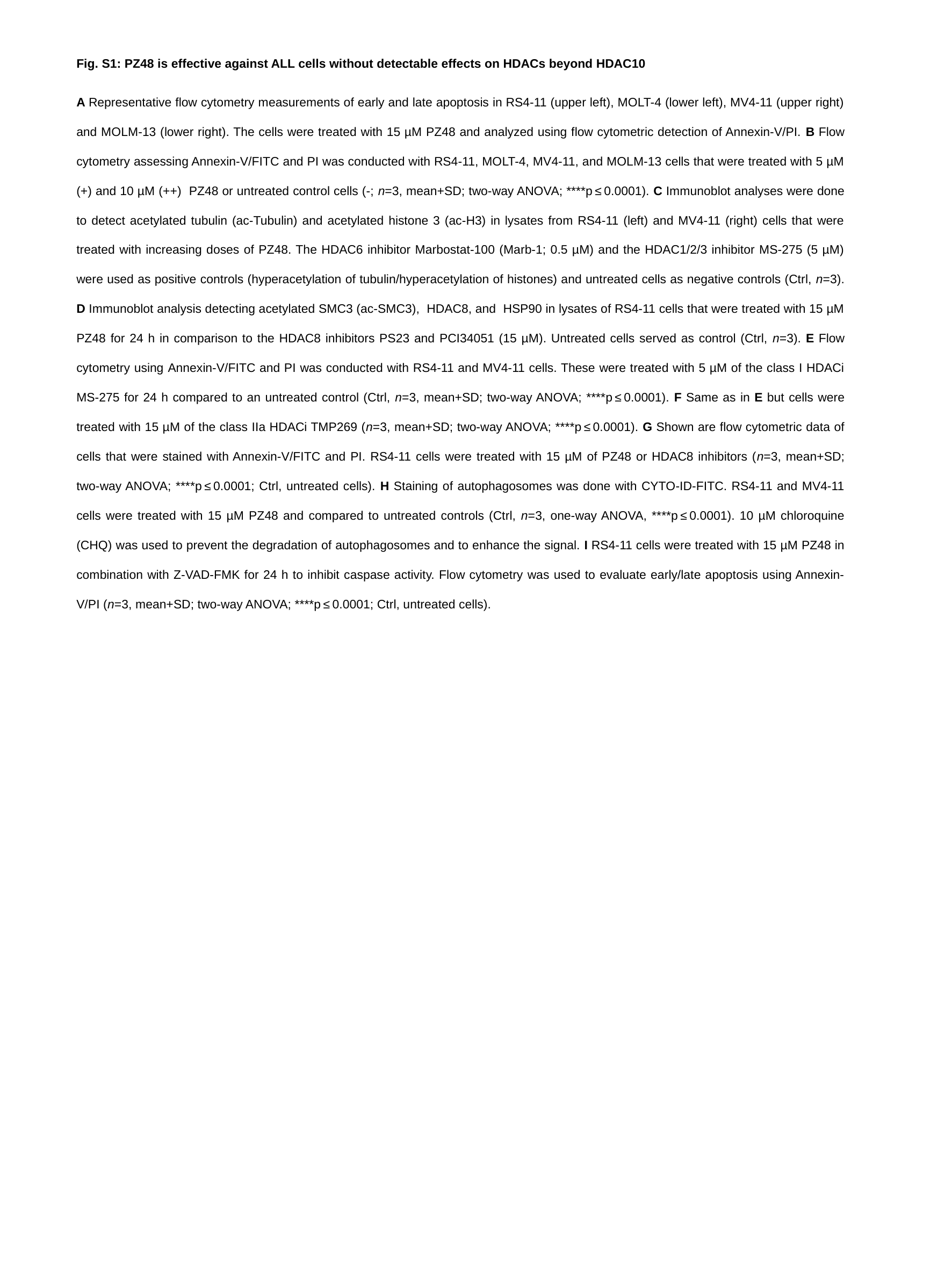

Fig. S1: PZ48 is effective against ALL cells without detectable effects on HDACs beyond HDAC10
A Representative flow cytometry measurements of early and late apoptosis in RS4-11 (upper left), MOLT-4 (lower left), MV4-11 (upper right) and MOLM-13 (lower right). The cells were treated with 15 µM PZ48 and analyzed using flow cytometric detection of Annexin-V/PI. B Flow cytometry assessing Annexin-V/FITC and PI was conducted with RS4-11, MOLT-4, MV4-11, and MOLM-13 cells that were treated with 5 µM (+) and 10 µM (++) PZ48 or untreated control cells (-; n=3, mean+SD; two-way ANOVA; ****p ≤ 0.0001). C Immunoblot analyses were done to detect acetylated tubulin (ac-Tubulin) and acetylated histone 3 (ac-H3) in lysates from RS4-11 (left) and MV4-11 (right) cells that were treated with increasing doses of PZ48. The HDAC6 inhibitor Marbostat-100 (Marb-1; 0.5 µM) and the HDAC1/2/3 inhibitor MS-275 (5 µM) were used as positive controls (hyperacetylation of tubulin/hyperacetylation of histones) and untreated cells as negative controls (Ctrl, n=3). D Immunoblot analysis detecting acetylated SMC3 (ac-SMC3), HDAC8, and HSP90 in lysates of RS4-11 cells that were treated with 15 µM PZ48 for 24 h in comparison to the HDAC8 inhibitors PS23 and PCI34051 (15 µM). Untreated cells served as control (Ctrl, n=3). E Flow cytometry using Annexin-V/FITC and PI was conducted with RS4-11 and MV4-11 cells. These were treated with 5 µM of the class I HDACi MS-275 for 24 h compared to an untreated control (Ctrl, n=3, mean+SD; two-way ANOVA; ****p ≤ 0.0001). F Same as in E but cells were treated with 15 µM of the class IIa HDACi TMP269 (n=3, mean+SD; two-way ANOVA; ****p ≤ 0.0001). G Shown are flow cytometric data of cells that were stained with Annexin-V/FITC and PI. RS4-11 cells were treated with 15 µM of PZ48 or HDAC8 inhibitors (n=3, mean+SD; two-way ANOVA; ****p ≤ 0.0001; Ctrl, untreated cells). H Staining of autophagosomes was done with CYTO-ID-FITC. RS4-11 and MV4-11 cells were treated with 15 µM PZ48 and compared to untreated controls (Ctrl, n=3, one-way ANOVA, ****p ≤ 0.0001). 10 µM chloroquine (CHQ) was used to prevent the degradation of autophagosomes and to enhance the signal. I RS4-11 cells were treated with 15 µM PZ48 in combination with Z-VAD-FMK for 24 h to inhibit caspase activity. Flow cytometry was used to evaluate early/late apoptosis using Annexin-V/PI (n=3, mean+SD; two-way ANOVA; ****p ≤ 0.0001; Ctrl, untreated cells).

## Slide 3
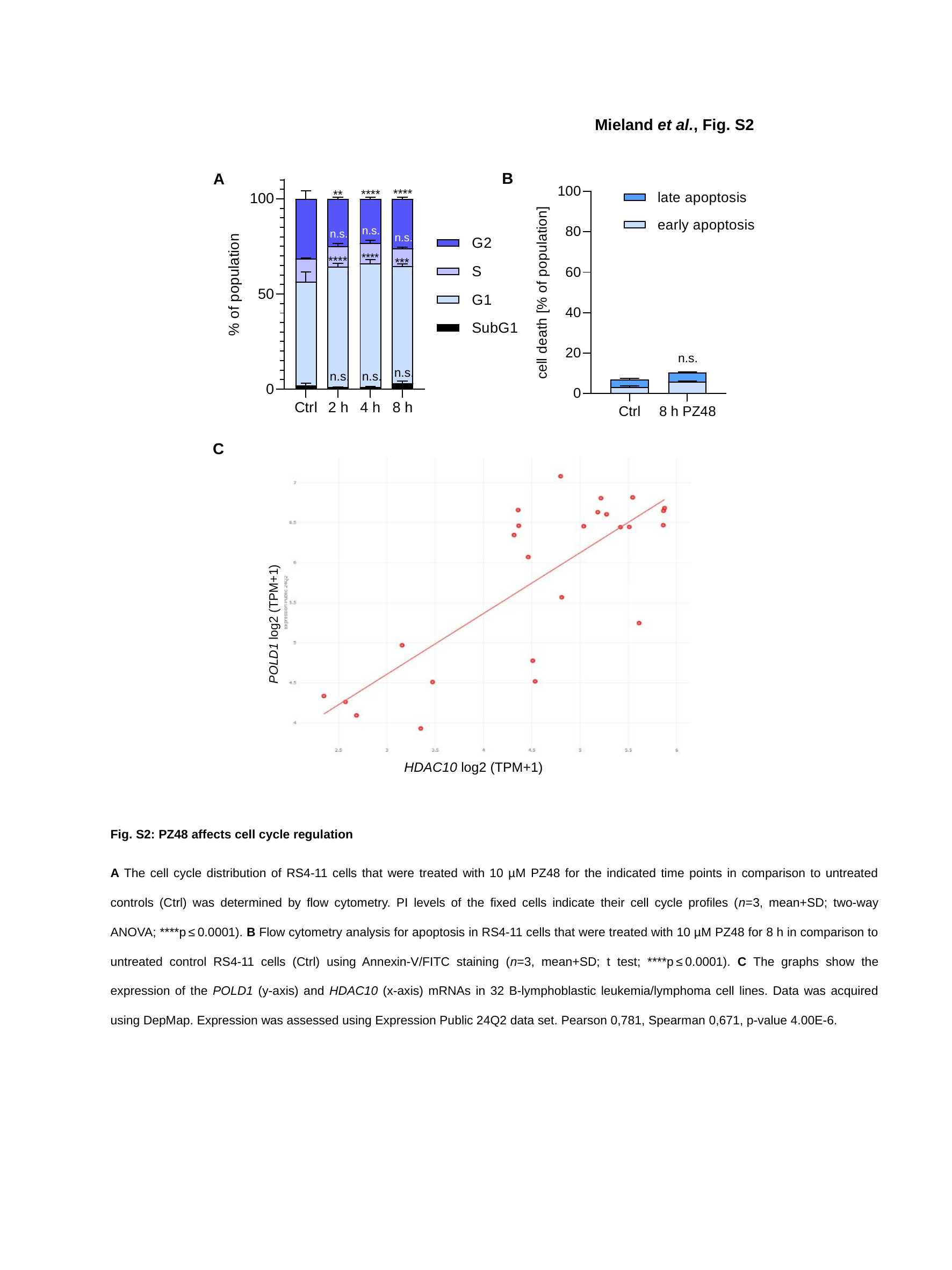

Mieland et al., Fig. S2
B
A
****
****
**
n.s.
n.s.
n.s.
****
****
***
n.s.
n.s.
n.s.
n.s.
C
POLD1 log2 (TPM+1)
HDAC10 log2 (TPM+1)
Fig. S2: PZ48 affects cell cycle regulation
A The cell cycle distribution of RS4-11 cells that were treated with 10 µM PZ48 for the indicated time points in comparison to untreated controls (Ctrl) was determined by flow cytometry. PI levels of the fixed cells indicate their cell cycle profiles (n=3, mean+SD; two-way ANOVA; ****p ≤ 0.0001). B Flow cytometry analysis for apoptosis in RS4-11 cells that were treated with 10 µM PZ48 for 8 h in comparison to untreated control RS4-11 cells (Ctrl) using Annexin-V/FITC staining (n=3, mean+SD; t test; ****p ≤ 0.0001). C The graphs show the expression of the POLD1 (y-axis) and HDAC10 (x-axis) mRNAs in 32 B-lymphoblastic leukemia/lymphoma cell lines. Data was acquired using DepMap. Expression was assessed using Expression Public 24Q2 data set. Pearson 0,781, Spearman 0,671, p-value 4.00E-6.

## Slide 4
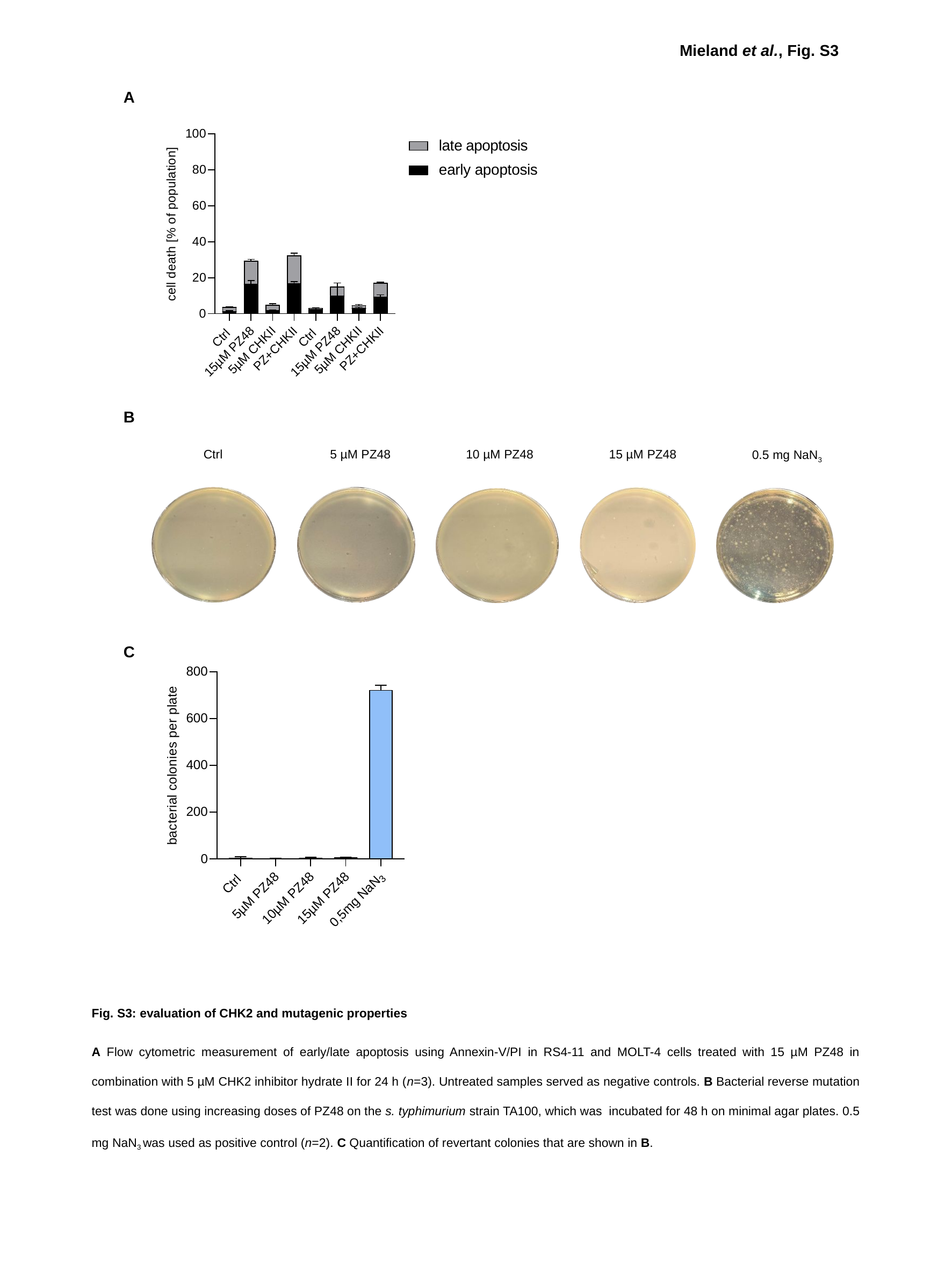

Mieland et al., Fig. S3
A
B
Ctrl
5 µM PZ48
10 µM PZ48
15 µM PZ48
0.5 mg NaN3
C
Fig. S3: evaluation of CHK2 and mutagenic properties
A Flow cytometric measurement of early/late apoptosis using Annexin-V/PI in RS4-11 and MOLT-4 cells treated with 15 µM PZ48 in combination with 5 µM CHK2 inhibitor hydrate II for 24 h (n=3). Untreated samples served as negative controls. B Bacterial reverse mutation test was done using increasing doses of PZ48 on the s. typhimurium strain TA100, which was incubated for 48 h on minimal agar plates. 0.5 mg NaN3 was used as positive control (n=2). C Quantification of revertant colonies that are shown in B.

## Slide 5
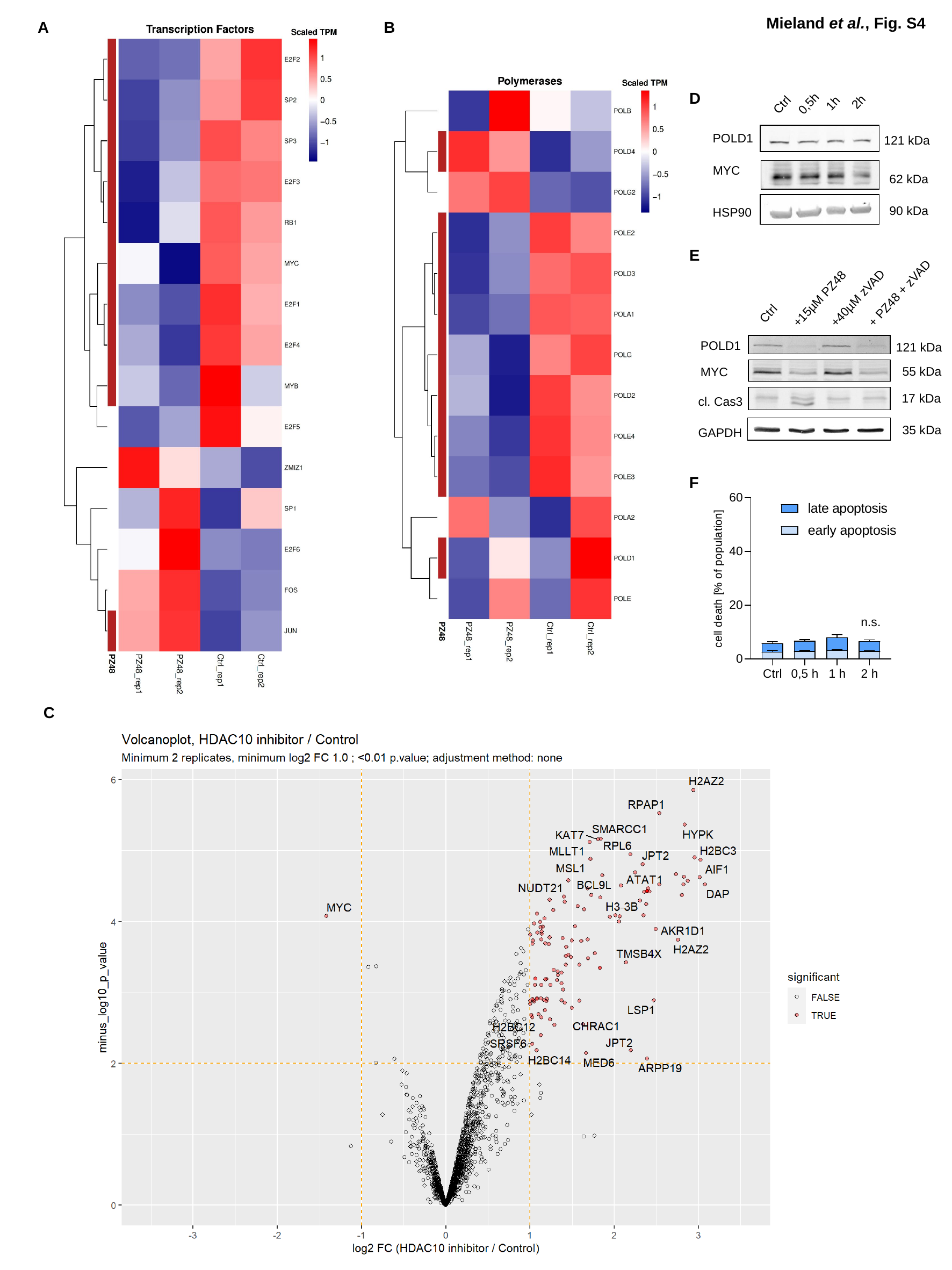

Mieland et al., Fig. S4
A
B
1h
Ctrl
0,5h
2h
D
POLD1
121 kDa
MYC
62 kDa
90 kDa
HSP90
+ PZ48 + zVAD
+40µM zVAD
Ctrl
+15µM PZ48
POLD1
121 kDa
55 kDa
MYC
17 kDa
cl. Cas3
35 kDa
GAPDH
E
F
n.s.
C

## Slide 6
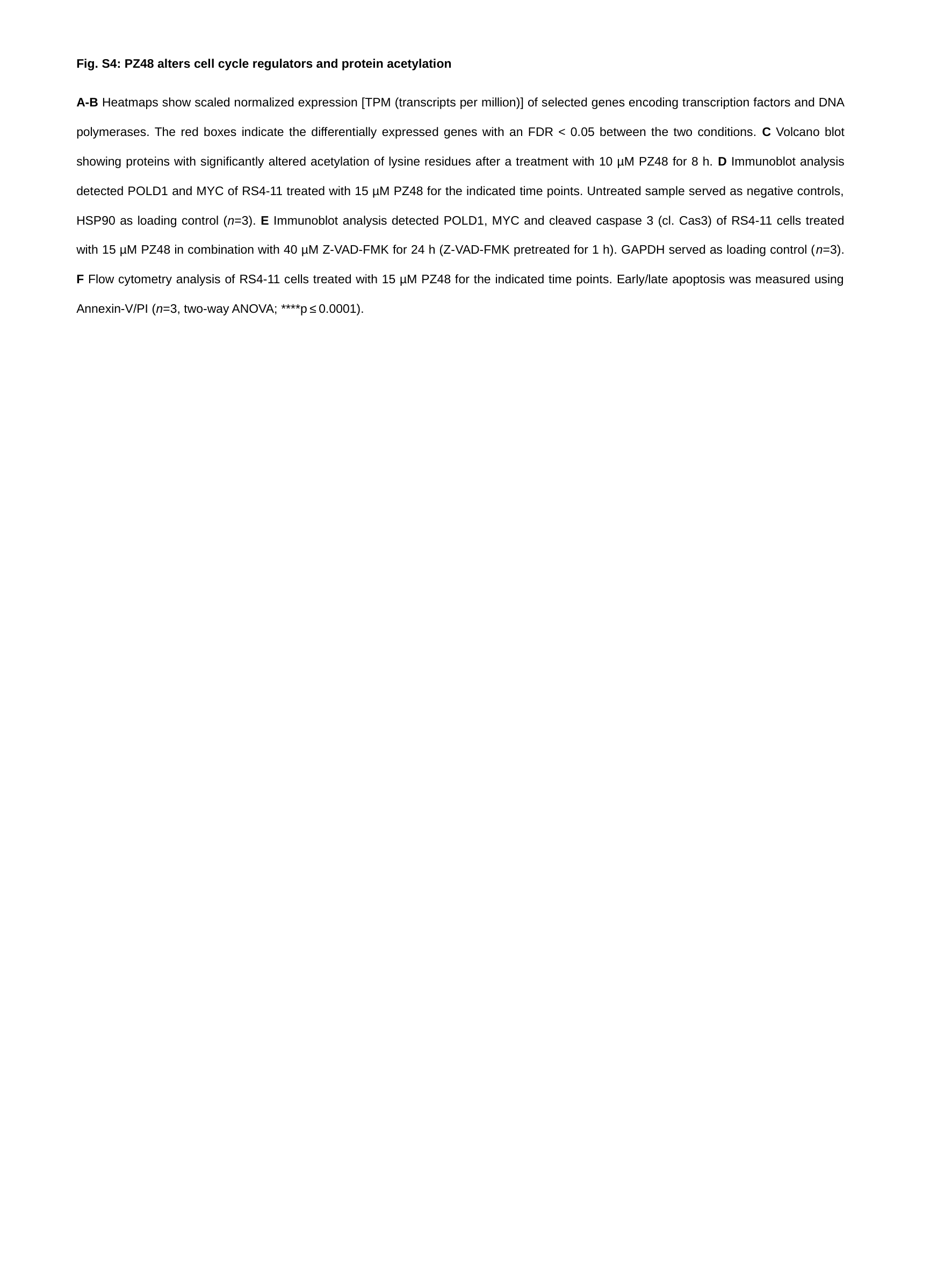

Fig. S4: PZ48 alters cell cycle regulators and protein acetylation
A-B Heatmaps show scaled normalized expression [TPM (transcripts per million)] of selected genes encoding transcription factors and DNA polymerases. The red boxes indicate the differentially expressed genes with an FDR < 0.05 between the two conditions. C Volcano blot showing proteins with significantly altered acetylation of lysine residues after a treatment with 10 µM PZ48 for 8 h. D Immunoblot analysis detected POLD1 and MYC of RS4-11 treated with 15 µM PZ48 for the indicated time points. Untreated sample served as negative controls, HSP90 as loading control (n=3). E Immunoblot analysis detected POLD1, MYC and cleaved caspase 3 (cl. Cas3) of RS4-11 cells treated with 15 µM PZ48 in combination with 40 µM Z-VAD-FMK for 24 h (Z-VAD-FMK pretreated for 1 h). GAPDH served as loading control (n=3). F Flow cytometry analysis of RS4-11 cells treated with 15 µM PZ48 for the indicated time points. Early/late apoptosis was measured using Annexin-V/PI (n=3, two-way ANOVA; ****p ≤ 0.0001).

## Slide 7
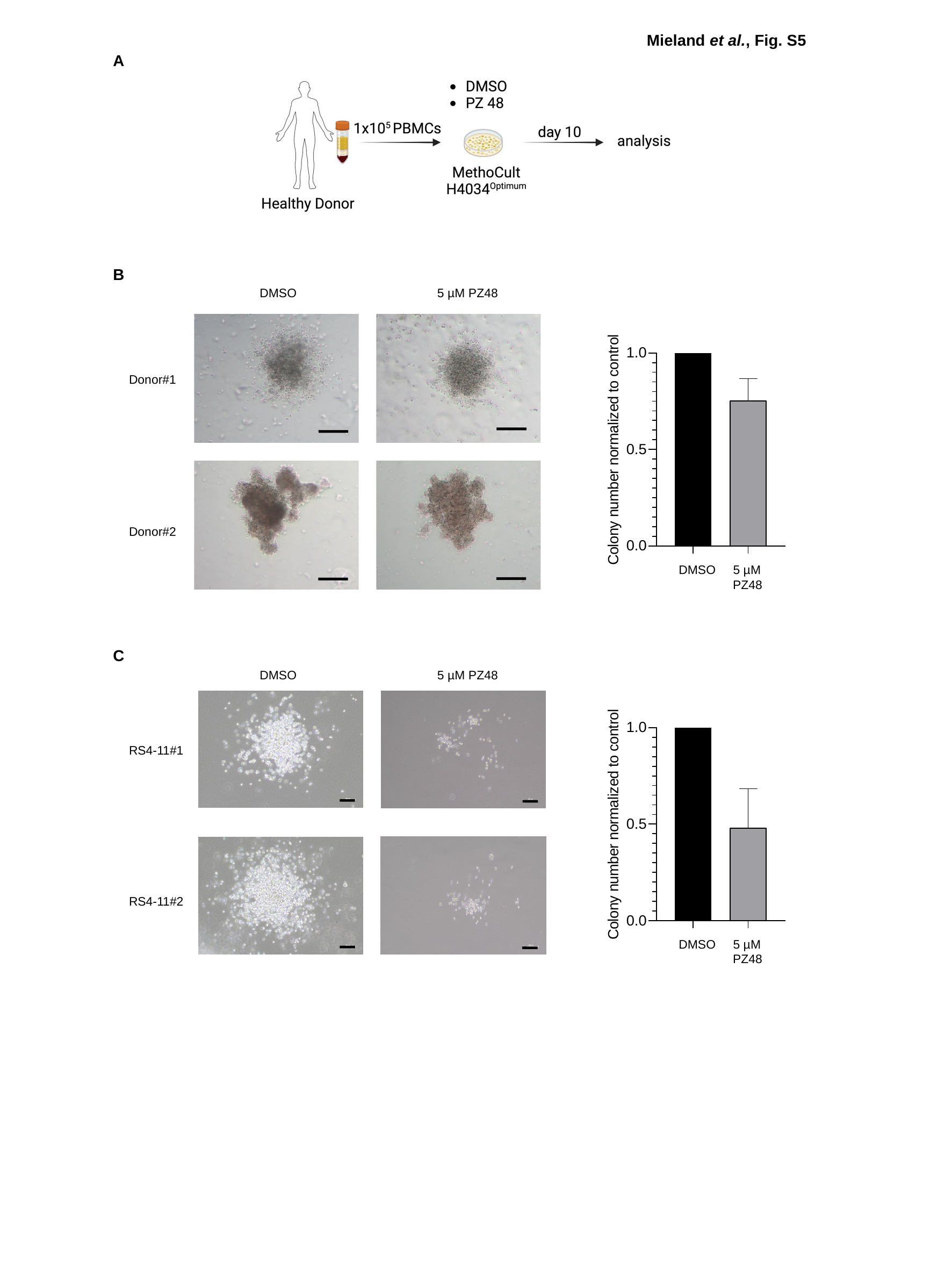

Mieland et al., Fig. S5
A
B
DMSO
5 µM PZ48
Donor#1
Donor#2
DMSO 5 µM 	 PZ48
C
DMSO
5 µM PZ48
RS4-11#1
RS4-11#2
DMSO 5 µM 	 PZ48

## Slide 8
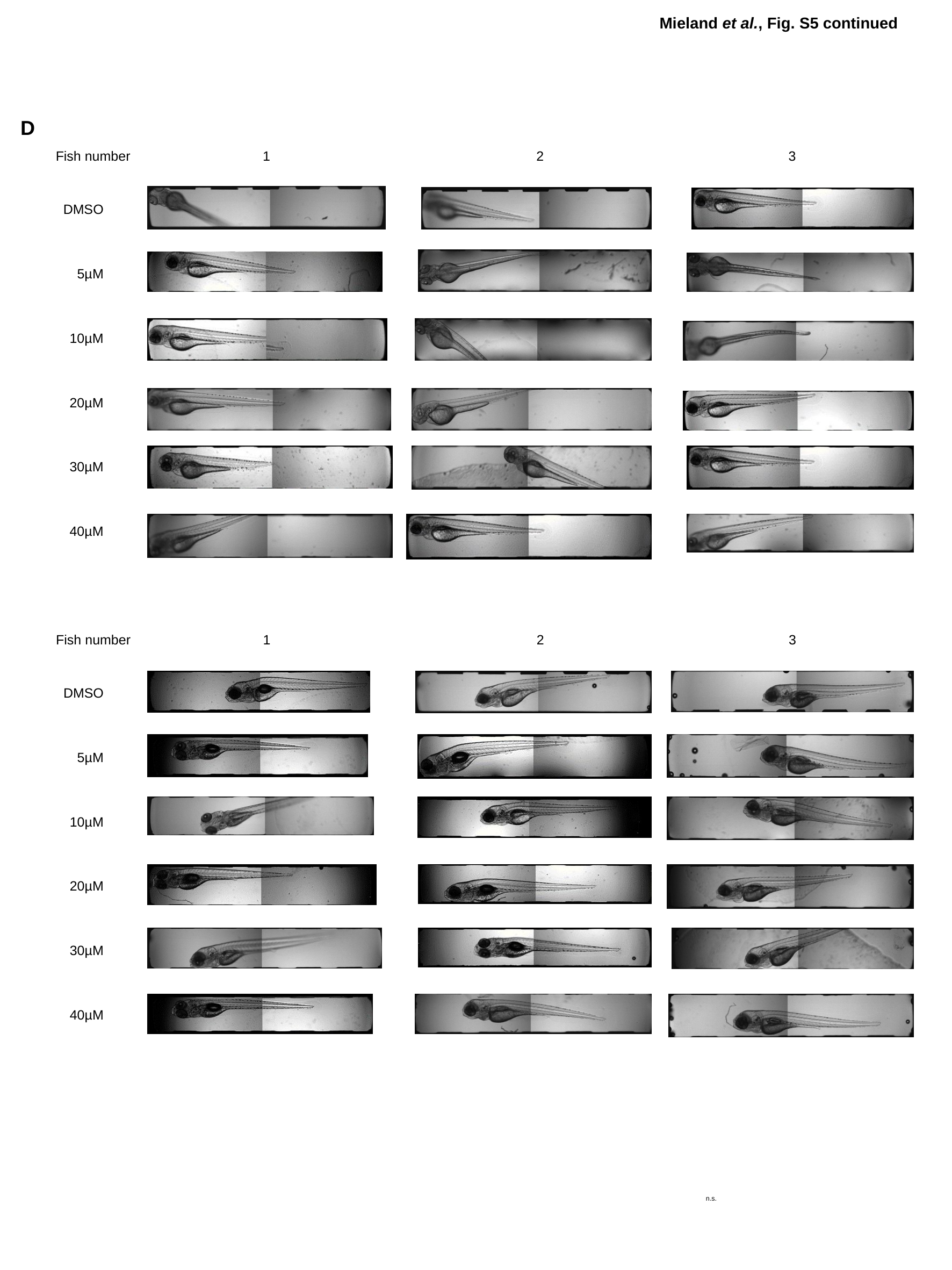

Mieland et al., Fig. S5 continued
D
Fish number 		 1 		 2 		 3
DMSO
5µM
10µM
20µM
30µM
40µM
Fish number 		 1 		 2 		 3
DMSO
5µM
10µM
20µM
30µM
40µM
n.s.

## Slide 9
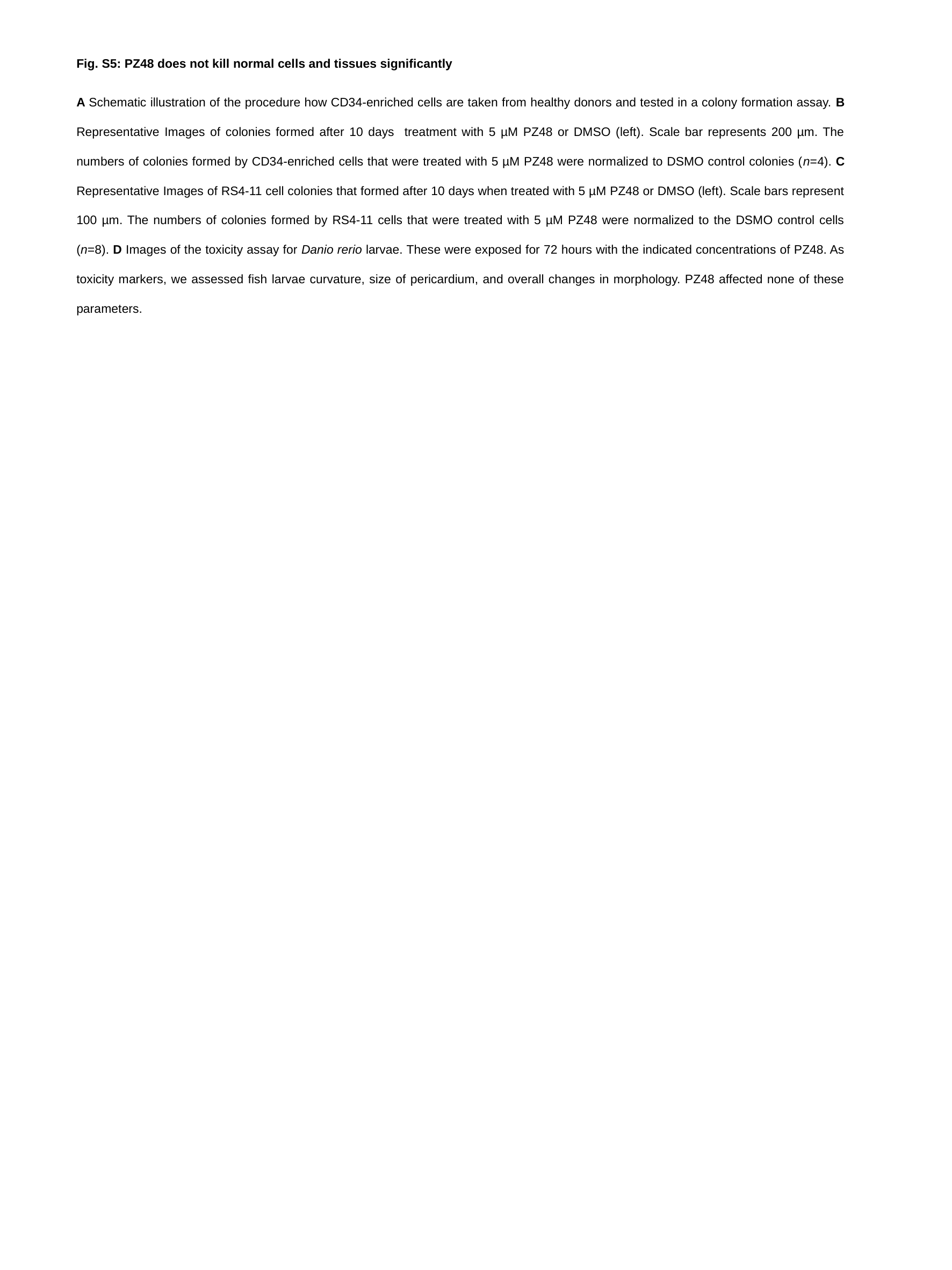

Fig. S5: PZ48 does not kill normal cells and tissues significantly
A Schematic illustration of the procedure how CD34-enriched cells are taken from healthy donors and tested in a colony formation assay. B Representative Images of colonies formed after 10 days treatment with 5 µM PZ48 or DMSO (left). Scale bar represents 200 µm. The numbers of colonies formed by CD34-enriched cells that were treated with 5 µM PZ48 were normalized to DSMO control colonies (n=4). C Representative Images of RS4-11 cell colonies that formed after 10 days when treated with 5 µM PZ48 or DMSO (left). Scale bars represent 100 µm. The numbers of colonies formed by RS4-11 cells that were treated with 5 µM PZ48 were normalized to the DSMO control cells (n=8). D Images of the toxicity assay for Danio rerio larvae. These were exposed for 72 hours with the indicated concentrations of PZ48. As toxicity markers, we assessed fish larvae curvature, size of pericardium, and overall changes in morphology. PZ48 affected none of these parameters.
