# Supplementary material for: The protein deacetylase HDAC10 controls DNA replication in malignant lymphoid cells
Source: Leukemia. 2025 Apr 29;39(7):1756–68. doi: 10.1038/s41375-025-02612-8 (PMC12208866; doi:10.1038/s41375-025-02612-8)
Supplement: Supplementary file 3 — Mieland et al Original blots [file 41375_2025_2612_MOESM3_ESM.pptx]

## Slide 1
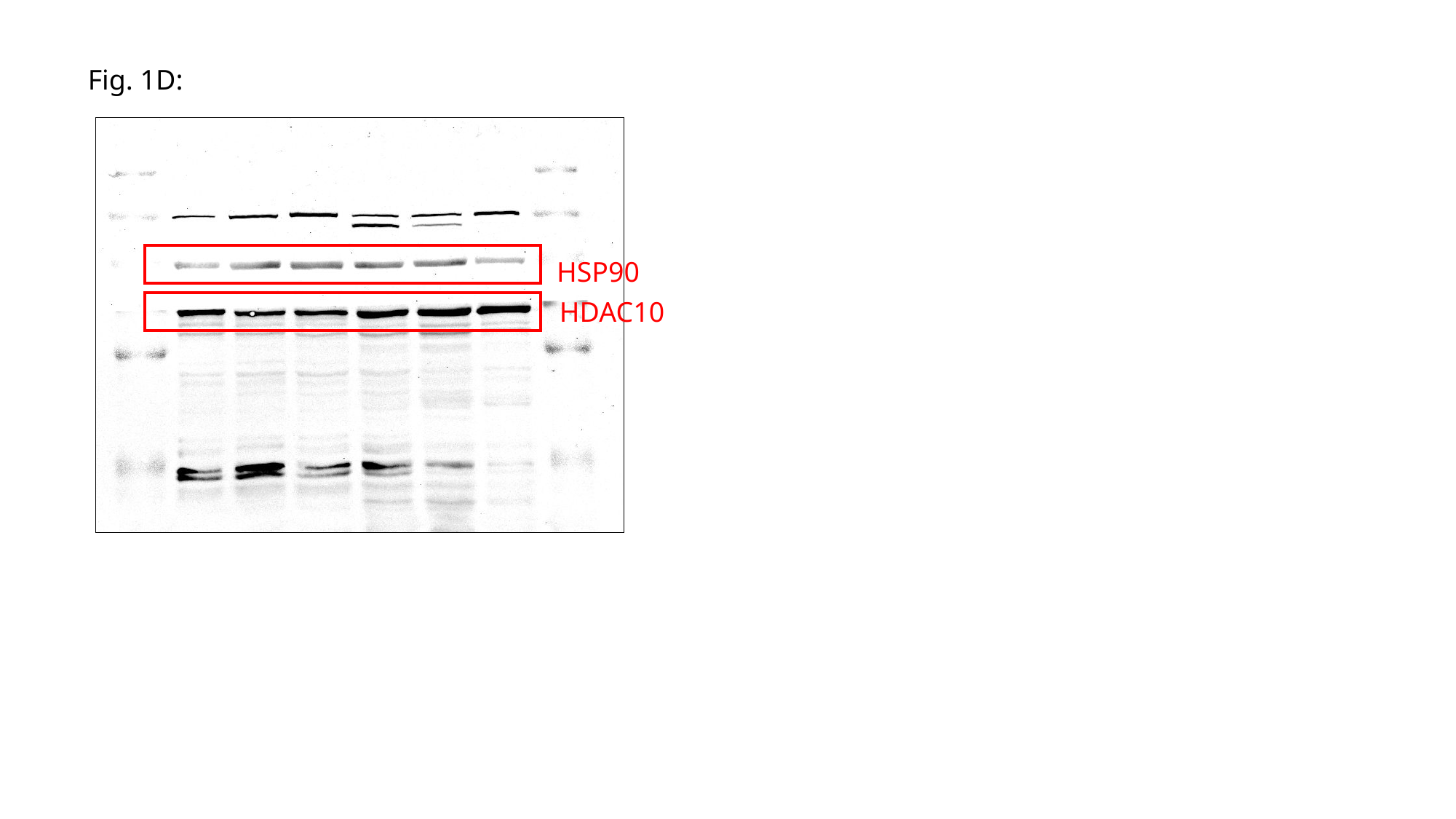

Fig. 1D:
HSP90
HDAC10

## Slide 2
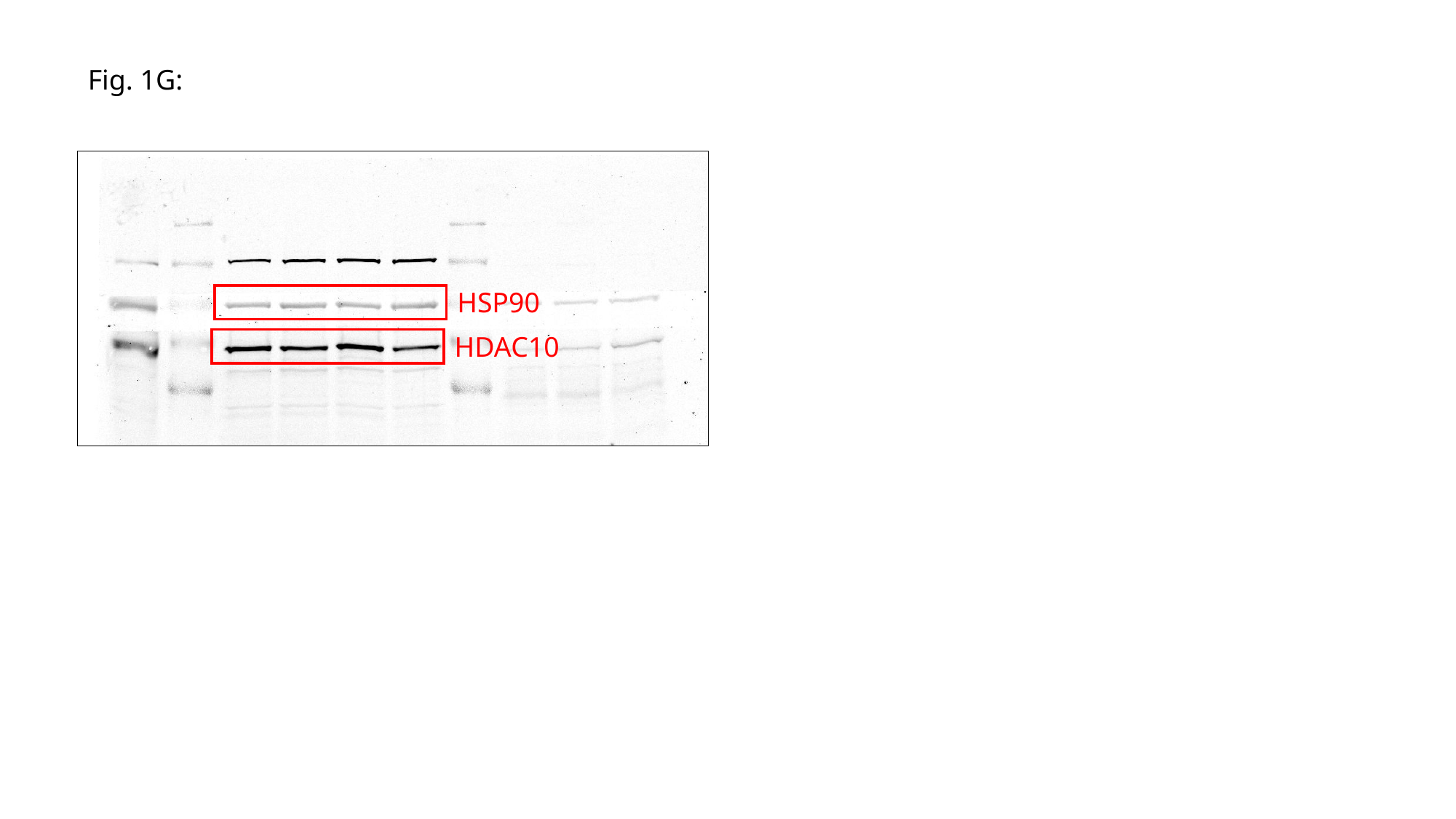

Fig. 1G:
HSP90
HDAC10

## Slide 3
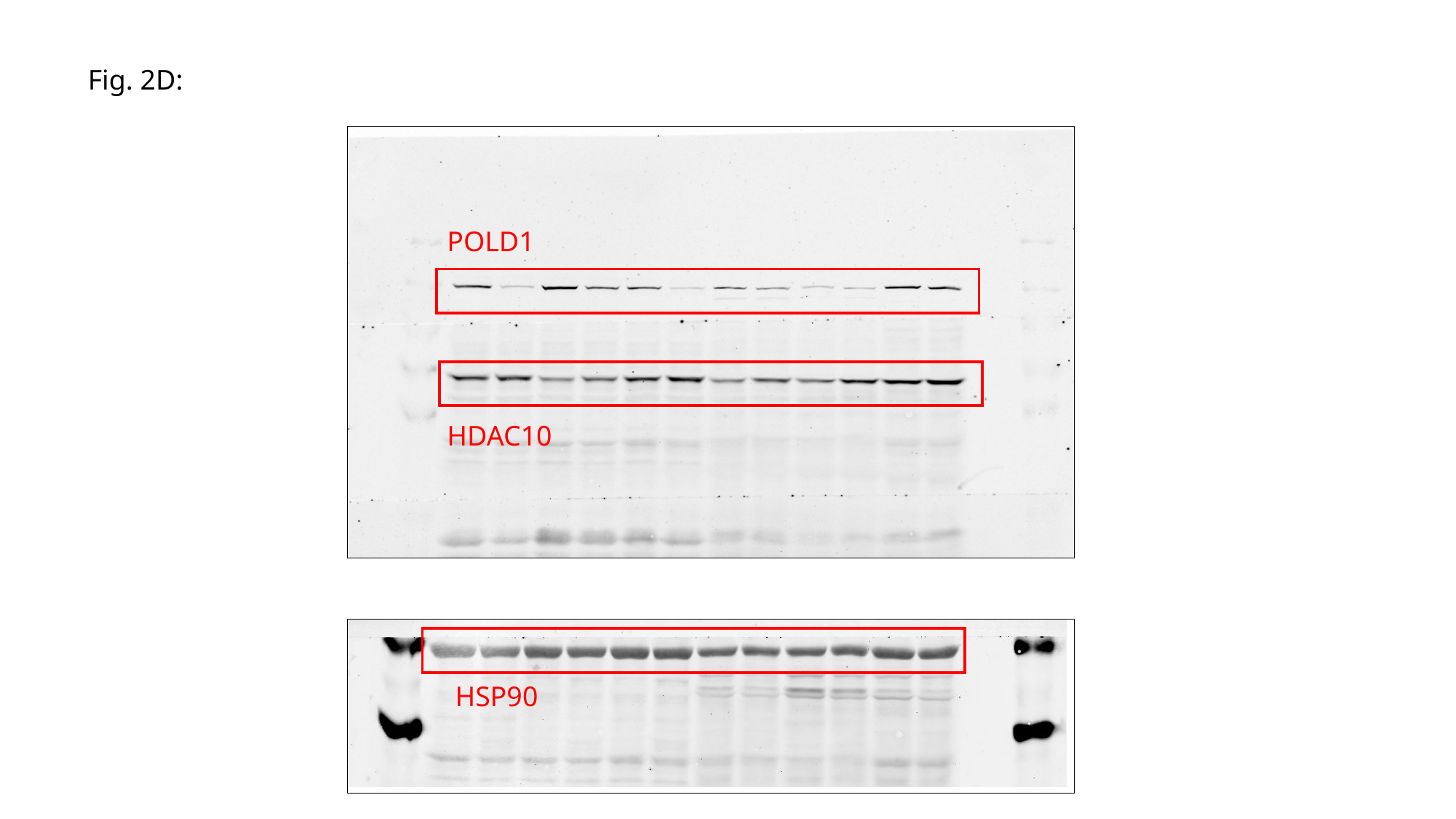

Fig. 2D:
POLD1
HDAC10
HSP90

## Slide 4
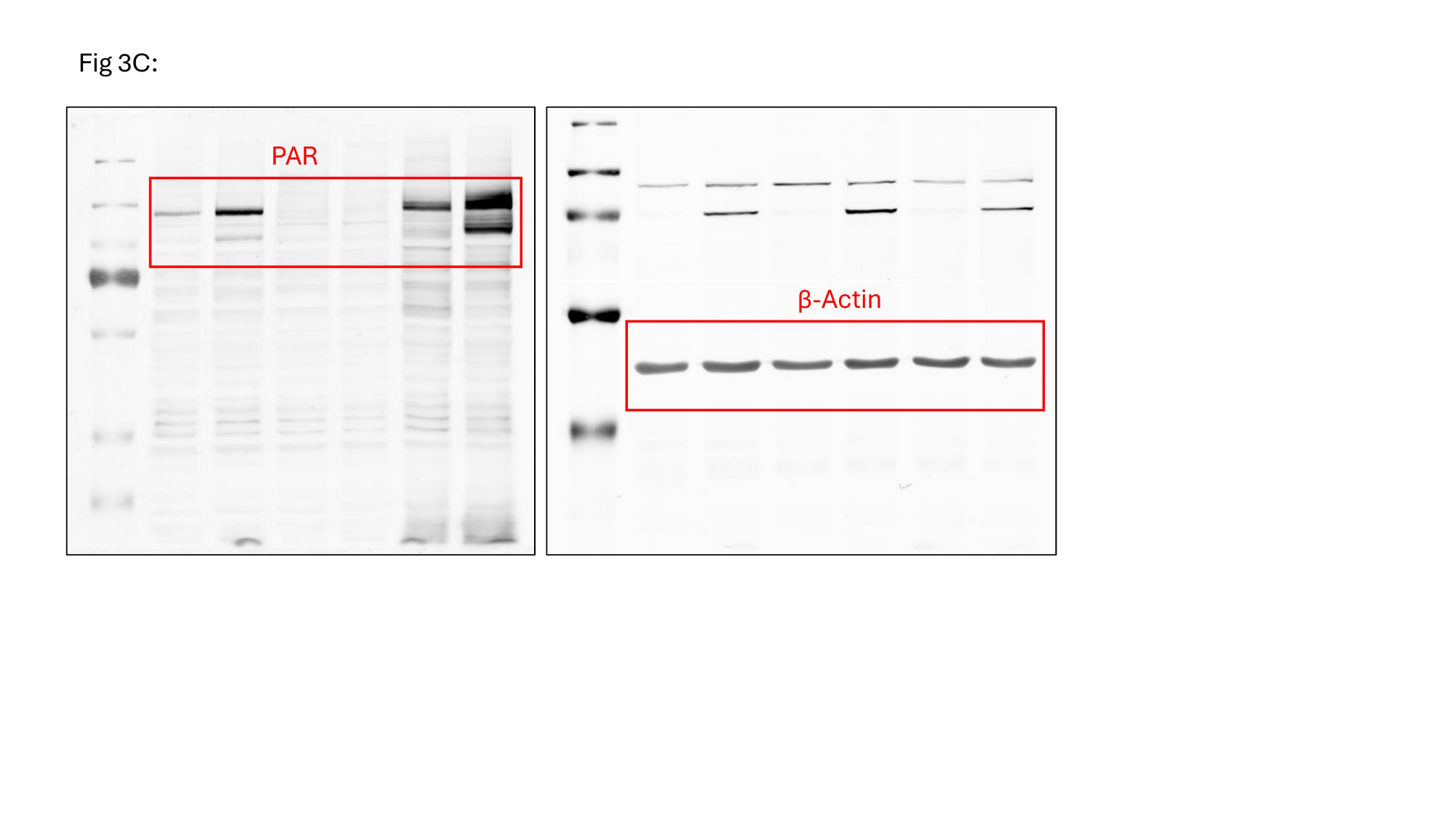

## Slide 5
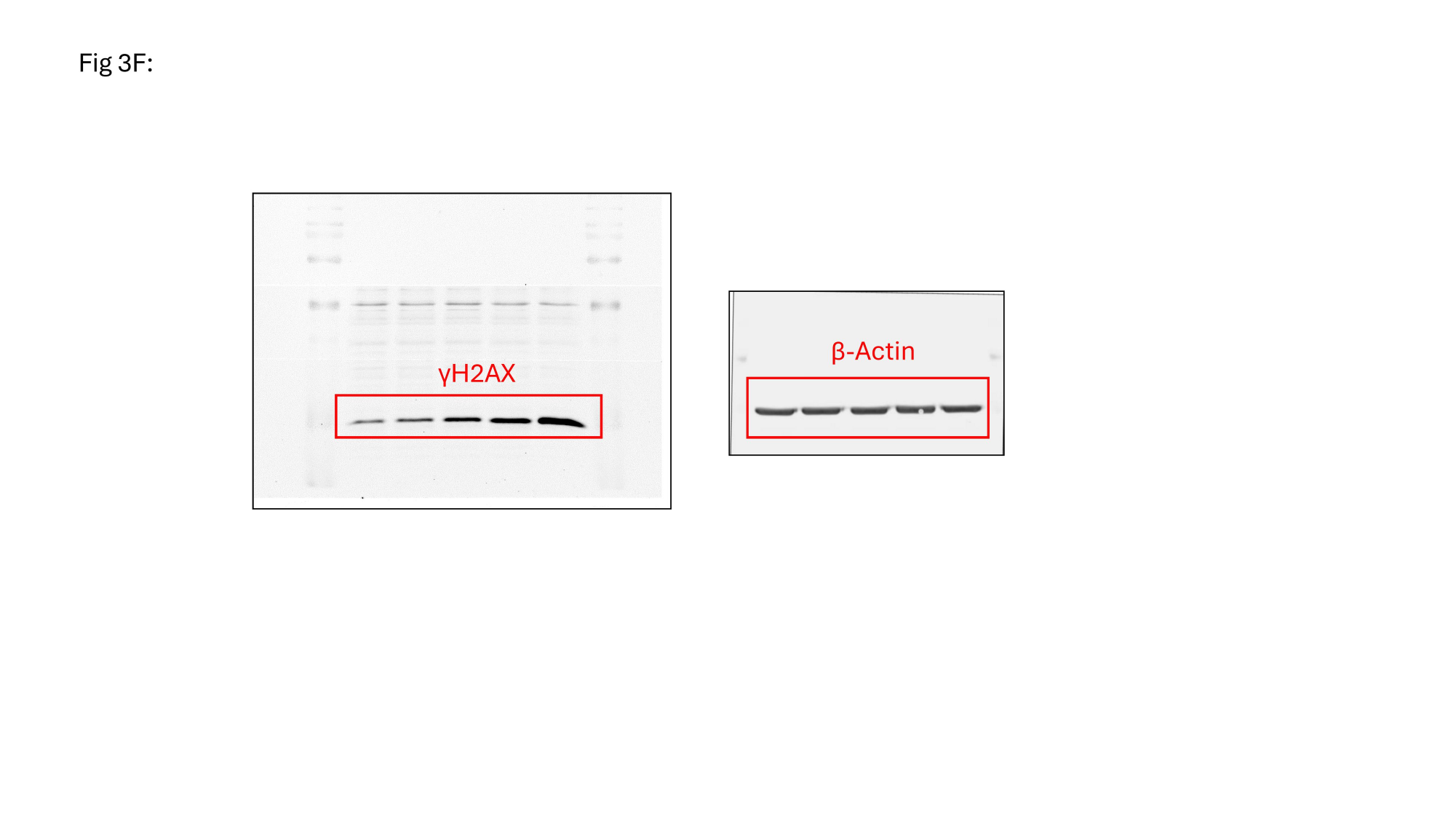

## Slide 6
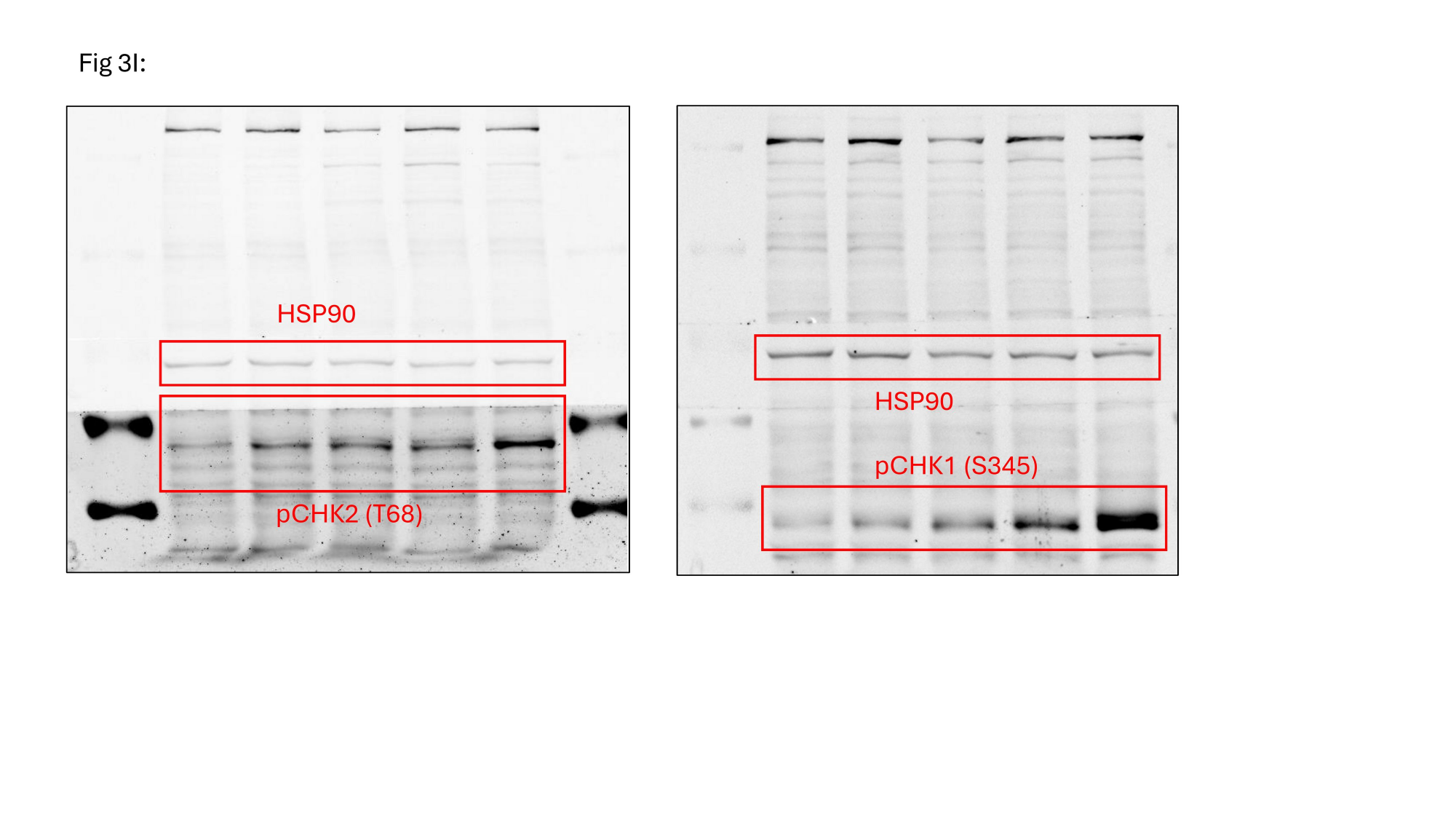

## Slide 7
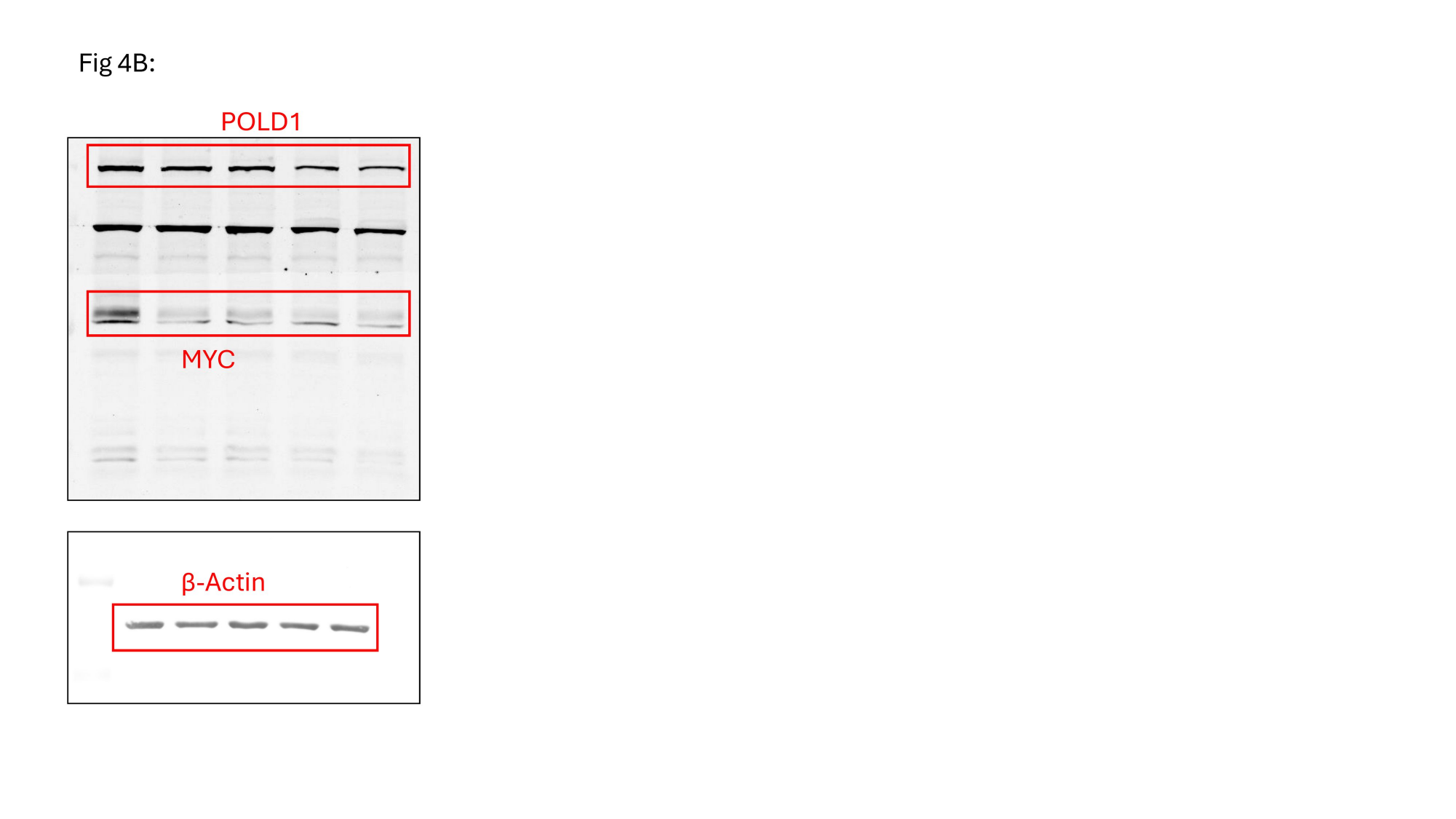

## Slide 8
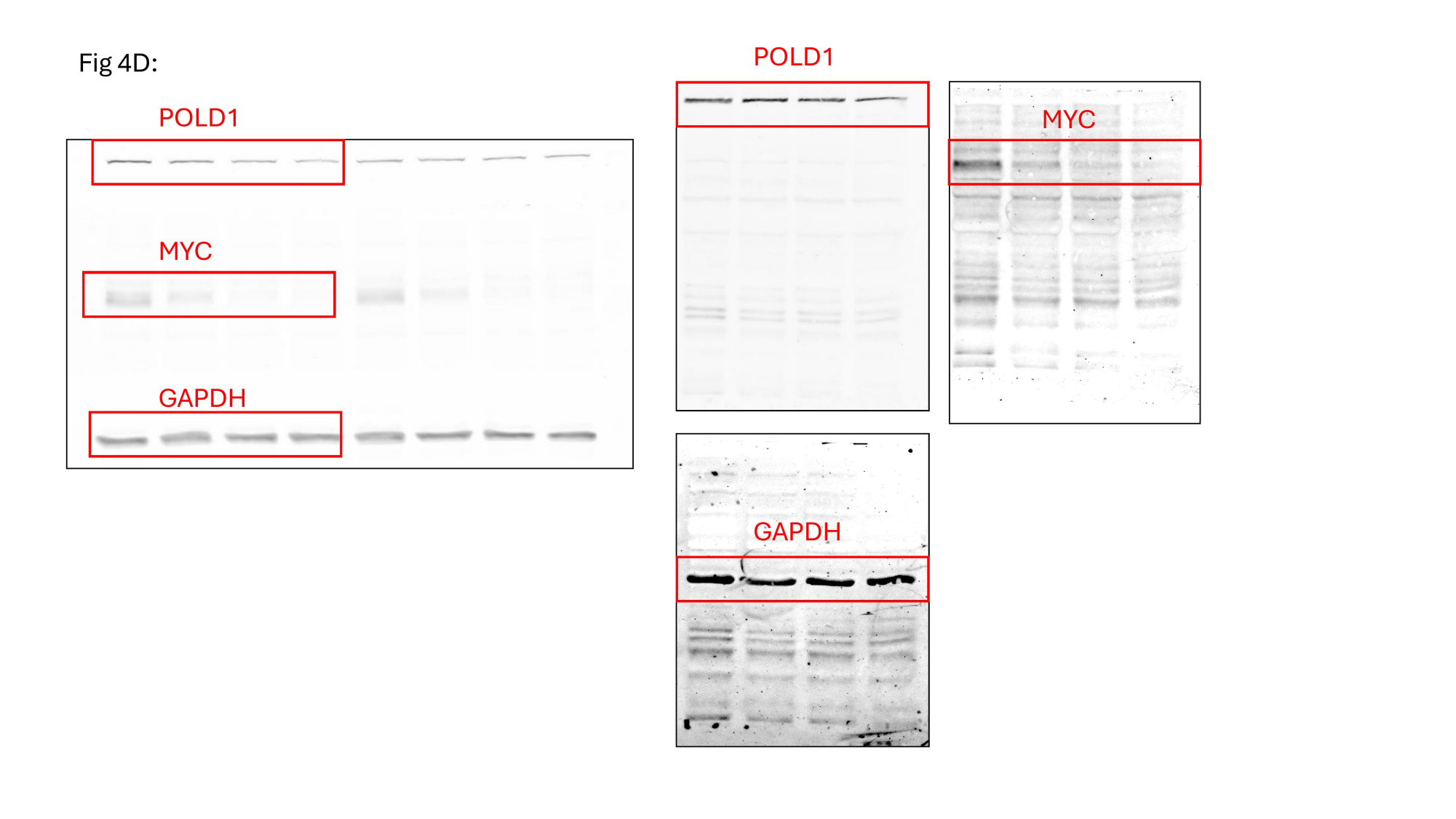

## Slide 9
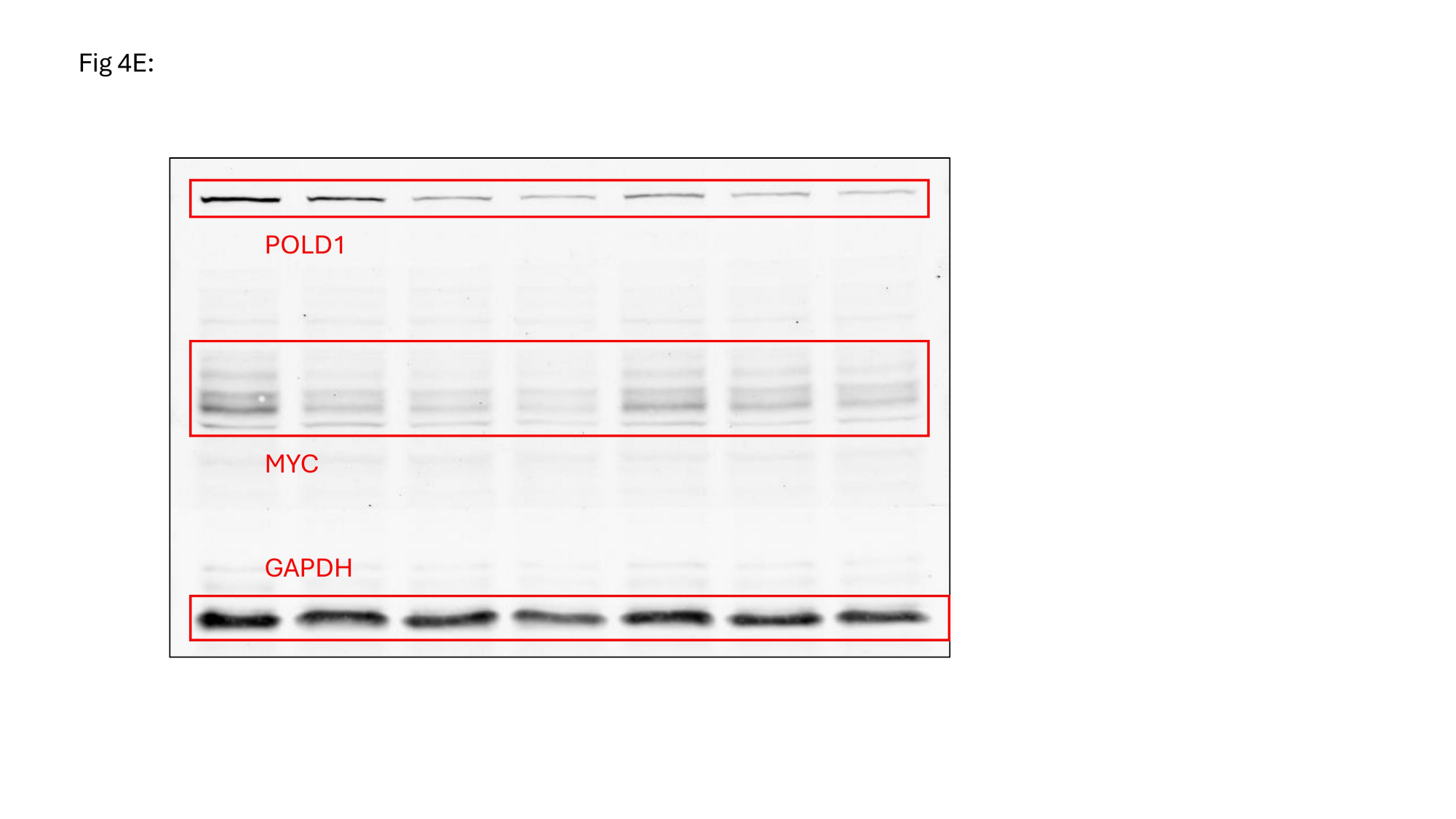

## Slide 10
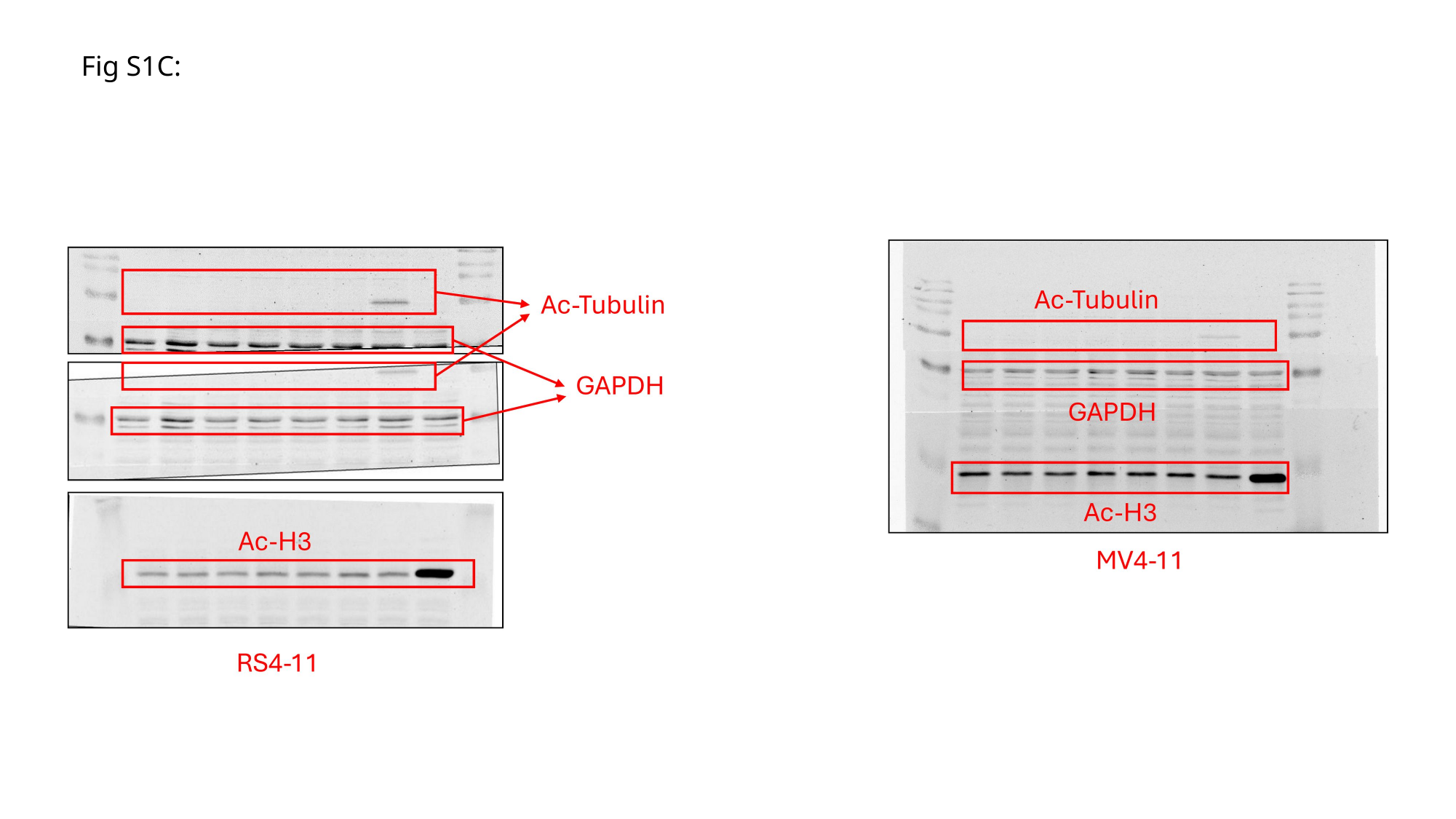

Fig S1C:

## Slide 11
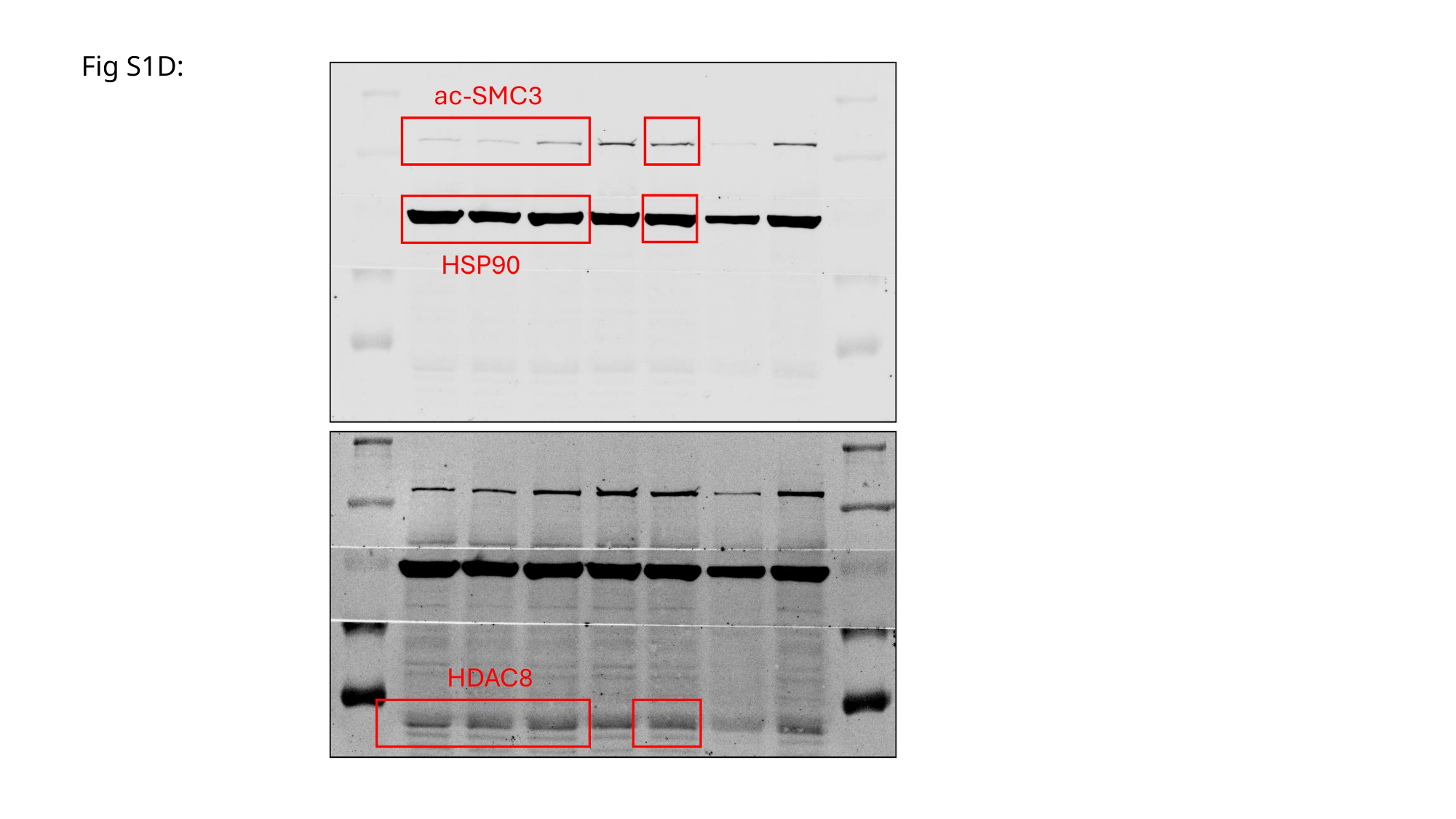

Fig S1D:

## Slide 12
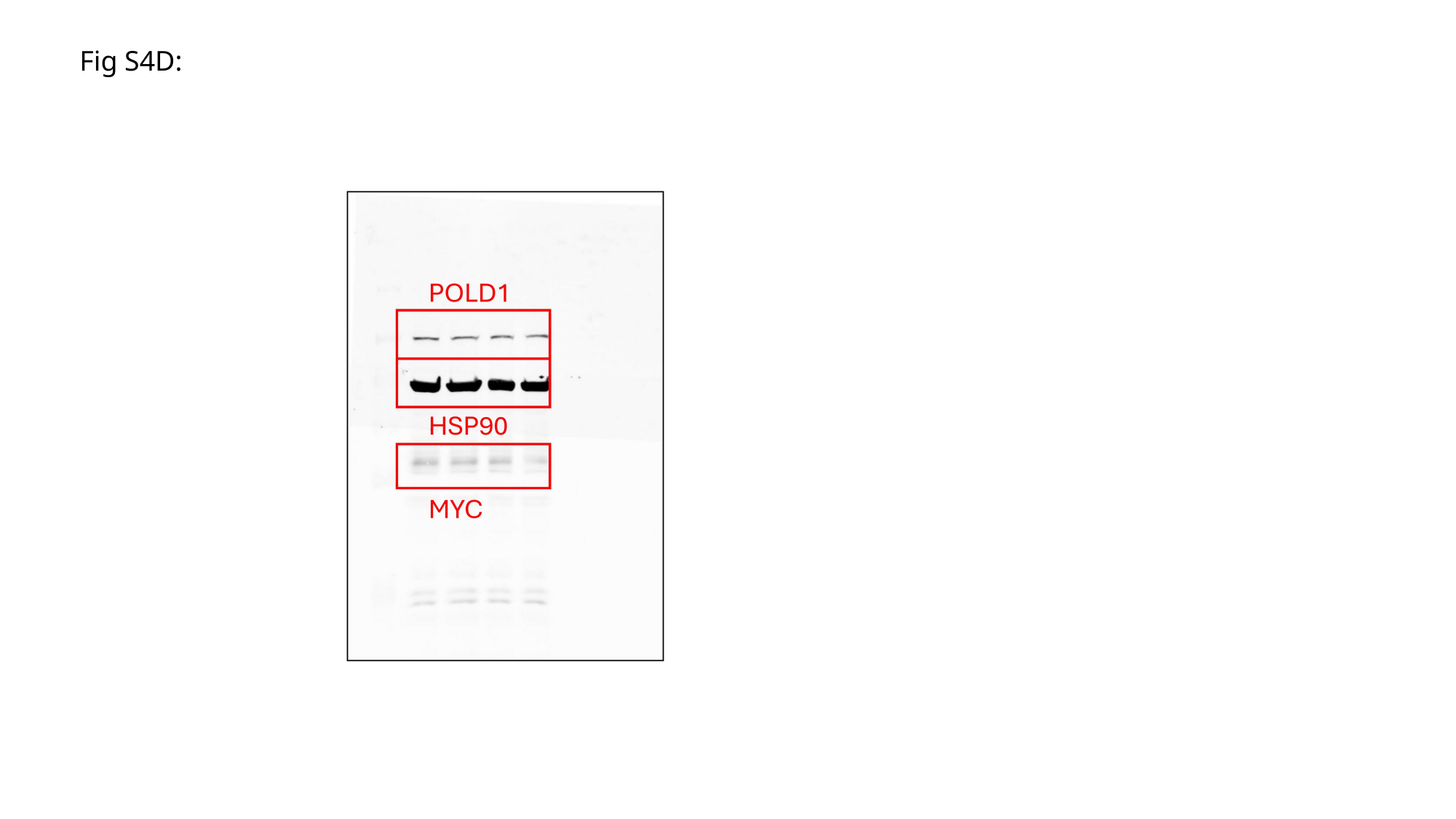

Fig S4D:

## Slide 13
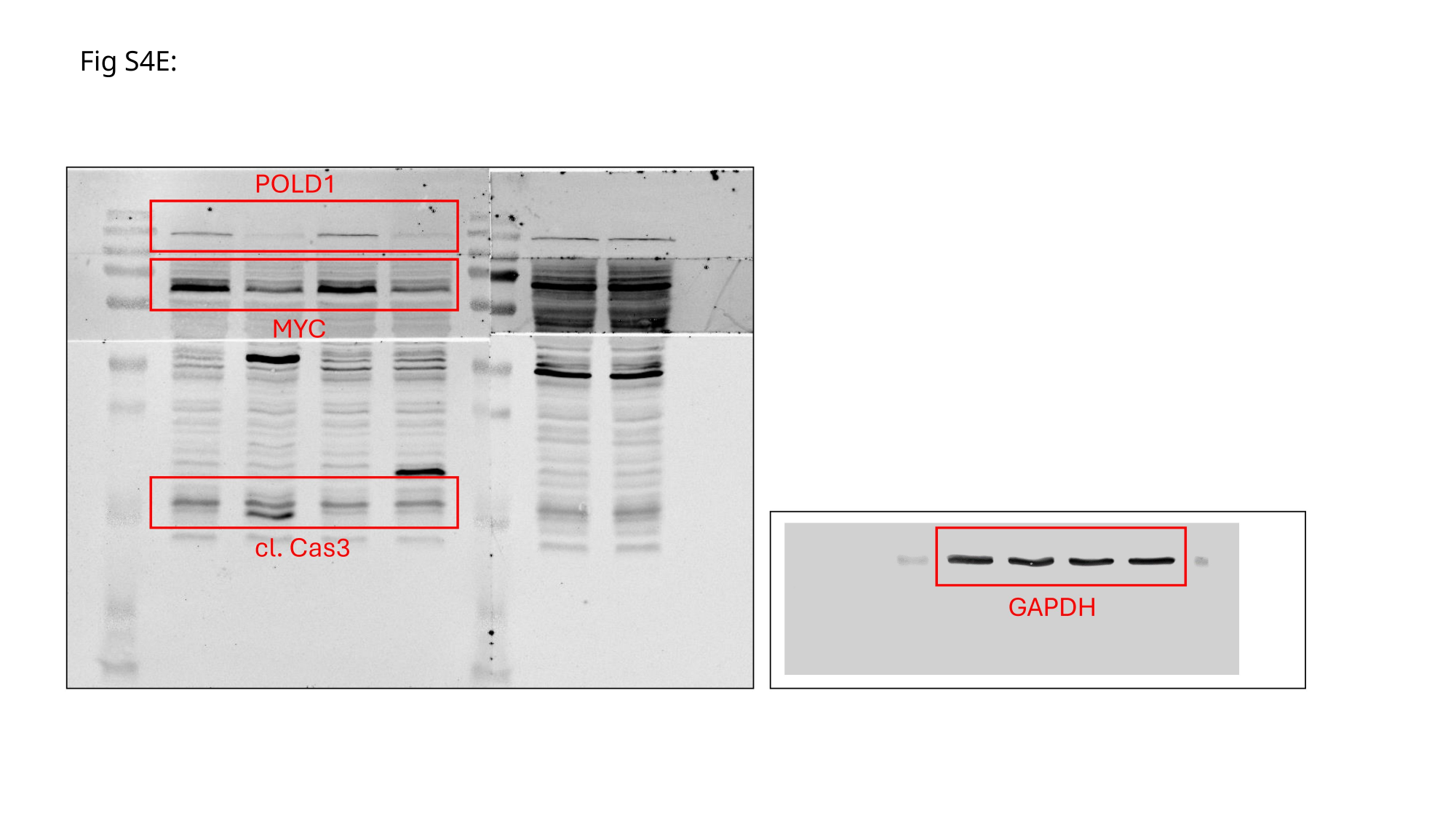

Fig S4E:
